# Comparison of Regular, Pure Shift, and Fast 2D NMR Experiments for Determination of the Geographical Origin of Walnuts

**DOI:** 10.3390/metabo11010039

**Published:** 2021-01-08

**Authors:** Stephanie Watermann, Caroline Schmitt, Tobias Schneider, Thomas Hackl

**Affiliations:** 1 Institute of Organic Chemistry, University of Hamburg, Martin-Luther-King-Platz 6, 20146 Hamburg, Germany; stephanie.watermann@chemie.uni-hamburg.de (S.W.); caroline.schmitt@chemie.uni-hamburg.de (C.S.); tobias.schneider@chemie.uni-hamburg.de (T.S.); 2 Hamburg School of Food Science—Institute of Food Chemistry, University of Hamburg, Grindelallee 117, 20146 Hamburg, Germany

**Keywords:** metabolomics, ASAP-HSQC, PSYCHE, multivariate statistics, walnut

## Abstract

^1^H NMR spectroscopy, in combination with chemometric methods, was used to analyze the methanol/acetonitrile (1:1) extract of walnut (*Juglans Regia* L.) regarding the geographical origin of 128 authentic samples from different countries (France, Germany, China) and harvest years (2016–2019). Due to the large number of different metabolites within the acetonitrile/methanol extract, the one-dimensional (1D) ^1^H NOESY (nuclear Overhauser effect spectroscopy) spectra suffer from strongly overlapping signals. The identification of specific metabolites and statistical analysis are complicated. The use of pure shift ^1^H NMR spectra such as PSYCHE (pure shift yielded by chirp excitation) or two-dimensional ASAP-HSQC (acceleration by sharing adjacent polarization-heteronuclear single quantum correlation) spectra for multivariate analysis to determine the geographical origin of foods may be a promising method. Different types of NMR spectra (1D ^1^H NOESY, PSYCHE, and ASAP-HSQC) were acquired for each of the 128 walnut samples and the results of the statistical analysis were compared. A support vector machine classifier was applied for differentiation of samples from Germany/China, France/Germany, and France/China. The models obtained by conduction of a repeated nested cross-validation showed accuracies from 58.9% (±1.3%) to 95.9% (±0.8%). The potential of the ^1^H-^13^C HSQC as a 2D NMR experiment for metabolomics studies was shown.

## 1. Introduction

The field of metabolomics is becoming increasingly important, especially with regard to the discrimination or classification of samples [1]. Food fingerprinting is a metabolomics-based approach that focuses on the recognition of specific patterns that enable the differentiation of several groups [1,2]. Regarding food samples, this differentiation can be based on the geographical origin, varieties, different growing conditions, adulterations, or harvest times [3,4,5,6,7]. The most common methods used for metabolomics studies are nuclear magnetic resonance spectroscopy (NMR) and mass spectrometry (MS), e.g., coupled to liquid chromatography (LC), gas chromatography (GC), or tandem MS [8,9,10]. Other methods include vibrational spectroscopy techniques such as infrared (IR) or Raman spectroscopy [11]. NMR spectroscopy requires minimal sample preparation and is a highly automatable technique, which therefore allows a high throughput of samples [8]. Furthermore, it enables structure elucidation, and it is a highly reproducible, nondestructive, and easily quantifiable method [8,9,10,12]. MS is much more sensitive compared to NMR spectroscopy, but signals in mass spectra are not directly quantifiable, since the ionization efficiency is not the same for different metabolites [8,9].

With regard to NMR based metabolomics, mostly one-dimensional (1D) ^1^H NMR spectra are used for following multivariate analysis [8,12]. Because of the very high isotopic natural abundance of ^1^H nuclei and their high sensitivity, the ^1^H NMR spectra show highest intensities compared to other types of NMR spectra such as ^13^C NMR spectra [8,12]. This high sensitivity leads to measuring times of only a few minutes, facilitating high throughput metabolomics studies [8]. However, since the spectra of complex metabolite mixtures contain up to hundreds of metabolites, the ^1^H NMR spectra severely suffer from spectral overlap [2,4]. Thus, the unambiguous identification of specific metabolites as well as the subsequent multivariate analysis gets complicated due to this lack of spectral resolution. Furthermore, less abundant metabolites cannot be detected at all when the peaks appear at resonances in the NMR spectra showing high concentrated metabolites [4].

An alternative is the acquisition of homonuclear decoupled ^1^H NMR spectra where homonuclear *J* couplings are collapsed into singlets and thus resulting in a higher spectral resolution [13,14]. The first fully homonuclear decoupled NMR spectrum was presented by Aue et al. in 1976 by a special projection of a two-dimensional (2D) *J*-resolved NMR spectrum [15]. In 1997, K. Zangger and H. Sterk developed a slice-selective experiment for homonuclear broadband-decoupling using a selective pulse during a weak pulsed field gradient [13,16]. This combination leads to a spatial dependent excitation along the NMR sample tube, where all signals of different frequencies are excited at once [16]. However, the overall signal intensity in the resulting spectrum is quite low because each signal is excited only in a small region of the NMR tube [16,17]. In 2014 the PSYCHE (pure shift yielded by chirp excitation) experiment was presented by Foroozandeh et al. making use of two low flip angle swept-frequency pulses during a weak pulsed field gradient [14,18]. The sensitivity of PSYCHE spectra is still quite low in comparison to regular ^1^H NMR spectra but, nevertheless, about tenfold better than the Zangger and Sterk method [18]. The PSYCHE spectra, in general, need to be acquired in a pseudo-2D manner [16,19]. Special data processing (data chunking technique) is required to produce the 1D pure shift NMR spectra [16,19]. This processing method, as well as the decoupling of strongly coupled protons, may lead to the occurrence of artefacts in the spectra [16]. An example of using PSYCHE spectra in combination with chemometrics for the analysis of adulterated honey samples and for the differentiation of tea samples from two different growing places was published by Bo et al. in 2019 [17]. Santacruz et al. performed a metabolomic study of soft corals using ^1^H NMR and PSYCHE spectra in combination with multivariate analysis for the correlation of their cytotoxic activity [20]. Lopez et al. analyzed fruits of the *Physalis peruviana* plant of different growing regions by using PSYCHE and a modification, namely the SAPPHIRE-PSYCHE (sideband averaging by periodic phase incrementation of residual *J* evolution) [21].

Another type of NMR experiment that yields a spectrum with higher resolution compared to 1D ^1^H NMR is the 2D ^1^H-^13^C HSQC (heteronuclear single quantum correlation) because the overlapping signals in the 1D ^1^H NMR spectra can be additionally resolved in the second ^13^C dimension along F1 [4]. Besides the improved resolution of the 2D ^1^H-^13^C HSQC spectra, additional information about the ^13^C resonances of the substances can be obtained without the need to acquire another spectrum. This additional information not only facilitates structure elucidation and assignment but is also suitable for multivariate analysis as the 2D peak intensities can be used directly as input variables [4]. The main drawback of using 2D NMR spectra for multivariate analysis is the long experiment time [22]. A possibility to circumvent this problem in the case of ^1^H-^13^C HSQC spectra is the utilization of the 2D ^1^H-^13^C ASAP-HSQC experiment (acceleration by sharing adjacent polarization) recently developed by Schulze-Sünninghausen et al. [22] relying on former studies about the ASAP-HMQC pulse sequence (heteronuclear multiple quantum-correlation) by Kupče et al. [23]. The ASAP-HSQC pulse sequence allows faster pulsing due to a reduced relaxation delay between subsequent scans without compromising in spectral quality [22]. In addition, in the ASAP-HSQC pulse sequence broadband shaped pulses such as BEBOP, BIBOP, and BURBOP shaped pulses derived from calculations of the Optimal Control Theory (OCT) can be used, which lead to a less amount of artifact signals in the resulting spectra [24]. Sharma et al. used 2D ASAP-HSQC spectra for the differentiation of black and green tea and compared the results with regular HSQC and ^1^H NMR spectra [4]. The studies of Le Guennec et al. included the evaluation of the general applicability of regular 2D HSQC spectra for metabolomics using synthetic samples [25]. Another study performed by Puig-Castellví et al. focused on the comparison of regular 2D HSQC and 1D ^1^H NMR spectra concerning the metabolism of yeast samples [26]. All studies showed that the use of 2D NMR spectra, especially HSQC spectra, seems to be a promising method both for multivariate analysis and for identification of metabolites.

In this study, classification models regarding the discrimination of the geographical origin (France/Germany/China) were built using the methanol/acetonitrile extract of 128 walnut samples. For each walnut sample, a 1D ^1^H NOESY spectrum, as well as a PSYCHE and an ^1^H-^13^C ASAP-HSQC spectrum, were acquired. The data was analyzed using multivariate statistics, including principal component analysis (PCA) and classification with a repeated nested cross-validation (CV) to avoid overfitting. By discrimination of samples from two countries, each using a linear support vector machine (SVM) classifier, high accuracies from 58.9% (±1.3%) to 95.9% (±0.8%) of the models were obtained. The resulting score and loading plots of the PCA, as well as the classification matrices of all types of NMR spectra, were compared among each other, and the feasibility of these types of NMR experiments for multivariate analysis was evaluated.

## 2. Results

**Figure 1:**
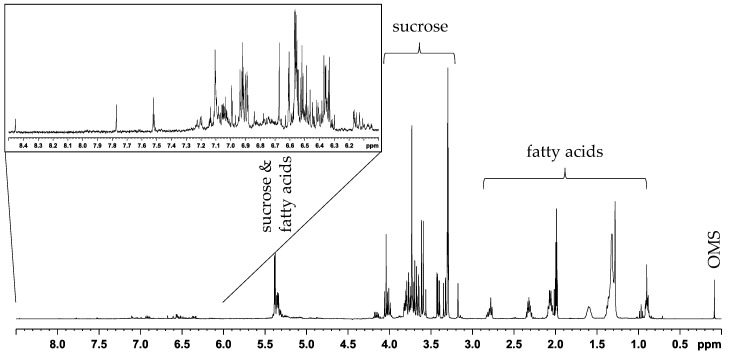
1D ^1^H NOESY spectrum (400 MHz) of walnut extract (acetonitrile-*d*_3_/methanol-*d*_4_, method A) showing signals from polar and non-polar compounds. The spectrum contains mainly signals from fatty acids. The region of carbohydrates is dominated by signals from sucrose. The region from 6.00 to 9.00 ppm is shown expanded.

The definition of an extraction method in a metabolomics-based approach is a crucial step as well as the choice of the experiment for data acquisition. Most studies regarding walnuts focus on the non-polar extract [27,28,29], and in the field of metabolomics, use of 1D NOESY NMR experiments with water suppression is established [30,31]. In previous studies, we analyzed the 1D NOESY spectra obtained by polar extraction with a mixture of buffer/methanol-*d*_4_/chloroform-*d* (5:4:6) [32]. Since the aim was to obtain a high dispersion in the chemical shift of NMR signals, the non-polar extract was unsuitable because the fatty acid fingerprint contains only a few characteristic signals. In this study, the mid-polar extract (acetonitrile-*d*_3_/methanol-*d*_4_) was used to obtain both signals from polar and non-polar metabolites. Furthermore, the extract was analyzed as an increased presence of secondary metabolites could be suspected. The extract exhibits many signals in the aromatic region, particularly below 7 ppm indicating polyphenols, and further signals in the region of carbohydrates. A representative 1D ^1^H NOESY spectrum of the acetonitrile-*d*_3_/methanol-*d*_4_ extract (method A) is shown in Figure 1.

The aliphatic region is dominated by signals from fatty acids. By far the most intense carbohydrate signals originate from sucrose. The signals in the aromatic region showed a much lower intensity compared to those from sucrose or the fatty acids. A stability measurement of the extract was carried out where it was observed that it is sufficiently stable. Only in the aromatic region some changes in signal intensity and signal shifts occur, which are shown in the supplementary information (Figure S1). The changes in signals of the extract were monitored for one week, whereby visible changes were mostly detected only after several days at room temperature. Consequently, the extract was sufficiently stable for the analytical purpose since changes in signals are only critical if they occur within few hours after extraction. In this study, a total of 128 walnut samples were used for discrimination of geographical origin.

### 2.1. PCA and Classification Using 1D ^1^H NOESY Spectra

**Figure 2:**
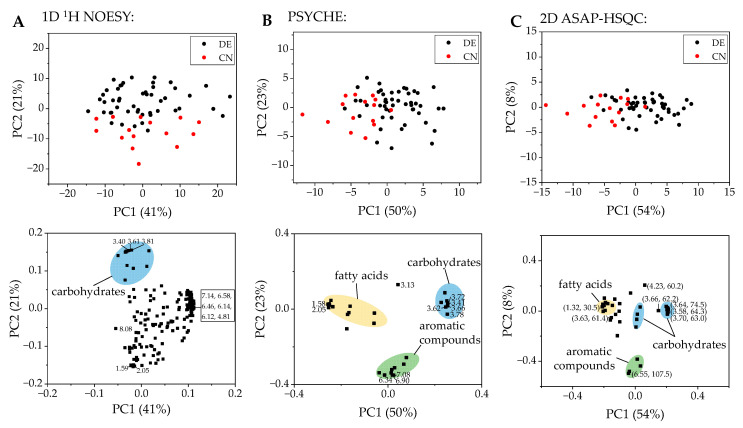
(**A**) PCA score plot of the differentiation of Chinese (CN) and German (DE) walnut samples using the 1D ^1^H NOESY spectra with the corresponding loading plot below. Explained variance: PC1 = 41%, PC2 = 21%. (**B**) PCA score plot using the PSYCHE spectra. The corresponding loading plot is shown below. Explained variance: PC1 = 50%, PC2 = 23%. (**C**) PCA score plot obtained by the ASAP-HSQC spectra with the corresponding loading plot below. Explained variance: PC1 = 54%, PC2 = 8%.

Walnut samples were weighed in triplicates to exclude preparation errors. The extraction was carried out according to method A and then spectra of the individual samples were measured by ^1^H NMR spectroscopy using the 1D ^1^H NOESY pulse sequence with water suppression. The spectra obtained were calibrated to OMS as reference and processed by automatic phase and baseline correction. The software Amix was used to visualize the data and to perform a PCA. After the analysis of the triplicates, no outliers were observed. For PCA, a real single spectrum (the first extract of each triplicate) was preferred instead of averaging the triplicates. A total of 183 buckets were manually defined for creating binary classification models. Additionally, the Kruskal-Wallis test as a non-parametric significance test was performed, revealing 44 buckets with a Bonferroni-corrected *p*-value below 0.00027322, indicating a high relevance in separation of the two sample groups. The buckets used with corresponding *p*-values are shown in the supporting material (Table S1). Surprisingly, only signals that were associated with fatty acids and carbohydrates showed significant differences. The PCA score and loading plot of DE/CN in Figure 2A, which were plotted using the software Origin, showed two partially overlapping clusters. The PCA score and loading plots of the differentiation France/Germany and France/China are shown in the supplementary information (Figures S2 and S3).

**Figure 3:**
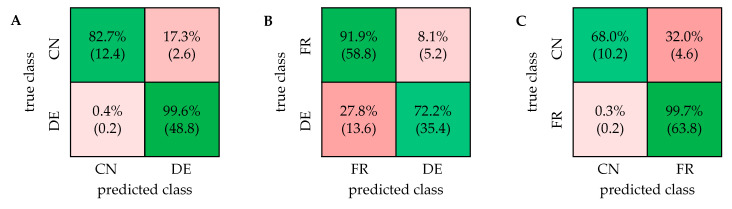
Confusion matrices of the discrimination of walnut samples from three different countries based on the 1D ^1^H NOESY spectra. CN: China; DE: Germany; FR: France. (**A**) Accuracy: 95.9% (±0.8%), (**B**) Accuracy: 83.4% (±2.0%), (**C**) Accuracy: 93.7% (±1.1%).

The classification was performed using the software Matlab with the normalized and scaled buckets as input. The Classification Learner App of Matlab includes the linear support vector machine classifier, which was used for all classifications with the same parameters. For validation, a repeated nested cross-validation (CV) was carried out. Conducting a nested CV, where each sample is left out exactly once for subsequent testing, produces results where the estimate of the true error is given almost unbiased [33]. Westerhuis et al. examined the validation of double CV as a variant of the nested CV by using permutation tests and showed that double CV leads to the most reliable results compared with other CV variants [34]. By repeating nested CV multiple times with different splits of the training and test sets, the influence of the split constitution can be further neglected [35]. For the repeated nested CV, the entire sample set was first separated into five parts, and one part was left out to function as the test set. The corresponding training set consists of the other four parts. A five-fold outer CV was carried out by repeating this procedure five times so that each walnut sample appeared once in a test set. The combination of the results obtained by each split presents the result as a confusion matrix. The whole procedure was performed with five randomly mixed splits to exhibit five combined confusion matrices. The result of a classification model was given as mean in a final confusion matrix. The accuracy is shown in percentage with the standard deviation. The classification was carried out using each possible combination of the three sample groups (DE/CN/FR), resulting in three classifiers. The final confusion matrices are presented in Figure 3. The linear SVM classifier led to the best results and therefore was used for all classification models.

The classification of German and Chinese walnut samples exhibited an accuracy of 95.9% (±0.8%), indicating a strong and robust model. Nearly all samples from Germany were correctly classified with an accuracy of 99.6%. The Chinese samples were classified correctly, with an accuracy of 82.7%. Misclassifications were mainly attributed to two Chinese samples, which showed a proximity to the cluster of the German samples in the PCA score plot. In the classification model based on the polar extract from previous studies, 5.3% of the fifteen Chinese samples were incorrectly classified [32]. The misclassification was attributed to only one sample, which was also classified wrongly in the model based on the mid-polar extract. For the differentiation of samples from France and Germany, the initially 183 selected buckets were used as well. The classification of samples from France and Germany resulted in a high accuracy of 83.4% (±2.0%). French samples were classified correctly with an accuracy of 91.9% and, in contrast, German samples with 72.2%. It was noticeable in the classification using this extraction method as well as the polar extraction from previous studies [32] that a relatively large number of samples from Baden-Württemberg were incorrectly classified, which can be explained by the proximity to France. In the classification based on the mid-polar extract, the proportion of incorrectly assigned samples with the origin Baden-Württemberg was 43%. In comparison, this value amounted to a similarly high value of approximately 57% for the classification with the polar extraction [32]. For the discrimination of samples from France and China, a high variability within each sample group was observed. An accuracy of 93.7% (±1.1%) was calculated and in total, nearly all samples from France were classified correctly, which corresponds to an accuracy of 99.7%. The Chinese samples were classified with an accuracy of 68.0%, which is probably due to the unequal sample size. The accuracy of the model based on the mid-polar extract was higher than the accuracy (92.6% (±1.2%)) obtained with the model based on the polar extract. In both extraction methods, misclassifications of Chinese samples were attributed to mainly the same samples. The 1D ^1^H NOESY spectra of the mid-polar extract of walnuts can be used to discriminate samples from different countries. In comparison with results from previous studies, it is noticeable that the accuracies of the models are similar when using the polar or mid-polar extract [32]. The results of both extraction methods are compared in the (supporting material Table S10).

### 2.2. Comparison with ASAP-HSQC and PSYCHE Spectra

**Figure 4:**
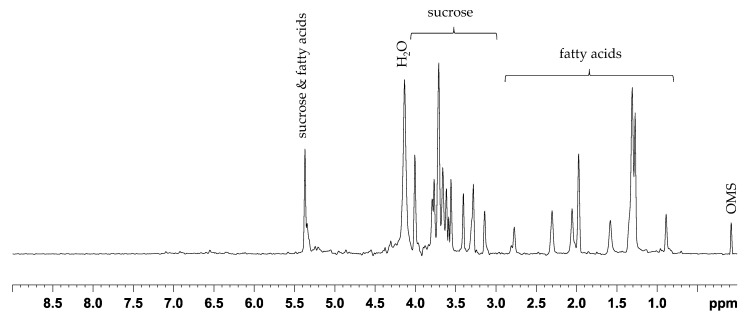
Representative PSYCHE spectrum (400 MHz) of the mid-polar walnut extract (acetonitrile-*d*_3_/methanol-*d*_4_ (method B)) showing the fatty acids in the aliphatic region as well as carbohydrates such as sucrose. Hardly any signals can be detected in the aromatic region of the spectrum.

For the comparison of the classification results concerning the geographical origin of the walnut samples using 1D ^1^H NOESY, ASAP-HSQC or PSYCHE spectra, the samples from Germany (49), France (64) and China (15) were additionally weighed in triplicates for acquisition of ASAP-HSQC and PSYCHE spectra. The samples were extracted using the extraction method B which is a slightly modified version of the acetonitrile/methanol extraction method A in order to achieve higher concentrations of the metabolites since the signal intensities in PSYCHE and ASAP-HSQC spectra are generally much lower compared to standard ^1^H NMR spectra. Figure 4 shows a homonuclear decoupled PSYCHE spectrum of a representative walnut sample.

The PSYCHE spectrum of the acetonitrile-*d*_3_/methanol-*d*_4_ extract (method B) in Figure 4 shows mostly good decoupling efficiency showing only singlets for chemically distinct NMR signals. In the aliphatic region signals from fatty acids were observed. Furthermore, signals from sucrose and the signal from water were detected. The overall signal intensity in the PSYCHE spectrum is quite low. Especially in the aromatic region hardly any signals were detected, and care must be taken to not consider artefacts in the baseline, originating from the special data processing technique, as small signals by mistake. Furthermore, the full width at half maximum (FWHM) of the peaks is rather large, resulting in just a slightly better resolution compared to regular ^1^H NMR spectra (compare Figure 1).

**Figure 5:**
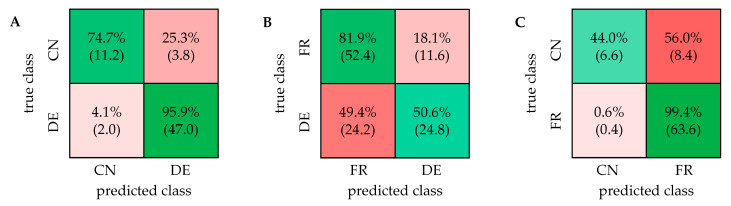
Confusion matrices for the classification of all two-class models based on the PSYCHE spectra. CN: China; DE: Germany; FR: France. Accuracies: (**A**) 90.9% (±1.5%); (**B**) 68.3% (±2.8%); (**C**) 88.9% (±1.2%).

For the visualization of the data, a PCA was performed using the software Amix. A total of 30 buckets were manually defined for all signals except the solvents and the reference (OMS) in the PSYCHE spectra (supplementary information S2). The corresponding score and loading plot are shown in Figure 2B. The results of the PCA for the differentiation of German and French as well as French and Chinese samples are shown in the supplementary information (Figures S2 and S3). The classification was performed similarly as for the 1D ^1^H NOESY spectra using a support vector machine algorithm in the Classification Learner App in Matlab. The composition of the training and test sets for the repeated nested CV was the same for all types of NMR experiments. The confusion matrices for all two-class models are shown in Figure 5.

In general, the results of the classification of different geographical origin of walnuts based on the PSYCHE spectra show great similarity to the results based on the 1D ^1^H NOESY spectra. The accuracy of the classification of German and Chinese samples is very high with 90.9% (±1.5%). The mainly misclassified samples are ca. three samples of Chinese origin and two samples of German origin, all within the overlapping region of the PCA score plot in Figure 2B. The result is very similar to the result of the ^1^H NOESY spectra since the misclassification of the Chinese samples mostly originates from the same samples. The classification accuracy of French and German samples was 68.3% (±2.8%). French samples were correctly classified with 81.9% accuracy, while half of the German samples were misclassified. On average, about 20 of the misclassified German walnut samples originate from south-west of Germany, namely from Baden-Württemberg and Hesse. This high misclassification therefore can be attributed to the geographical proximity of those specific walnut samples to France. The classification of French and Chinese samples shows an accuracy of 88.9% (±1.2%). While French samples are classified correctly with 99.4% most of the Chinese samples are misclassified, which can be attributed to the unequal sample sizes.

**Figure 6:**
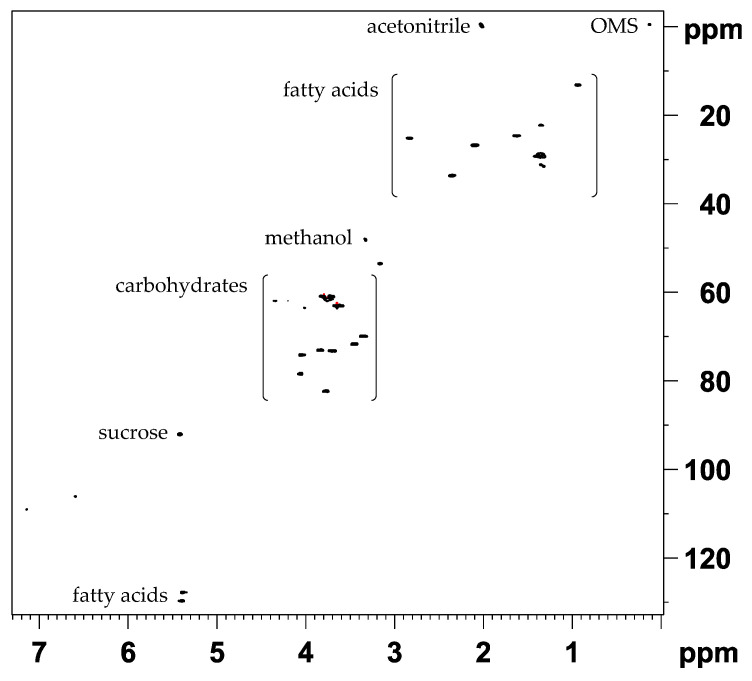
Representative 2D ASAP-HSQC spectrum (400 MHz) of a walnut sample extracted with acetonitrile-*d*_3_/methanol-*d*_4_ (method B) showing mainly signals of fatty acids and carbohydrates. Due to the additional ^13^C dimension, several signals occur less overlapping than in 1D NMR spectra.

For further enhancement of the spectral resolution regarding the overlap of signals, the acquisition of 2D NMR spectra seems to be promising. Due to the additional ^13^C frequency dimension in 2D ^1^H-^13^C HSQC spectra overlapping signals in the ^1^H frequency domain can partially be distinguished more clearly. Since the total acquisition time of 2D NMR spectra in general is much longer compared to 1D NMR data, techniques for the reduction of the measuring time need to be employed, especially regarding high throughput metabolomics studies. Thus, the ASAP-HSQC sequence developed by Schulze-Sünninghausen et al. was used by which the relaxation delay between the scans can be reduced without affecting the quality of the spectra [22]. Furthermore, the ASAP-HSQC pulse sequence was used since the applied pulses derived from the Optimal Control Theory seem to lead to artifact-free spectra [24]. However, the obtained ASAP-HSQC spectra are, in fact, quite similar to regular HSQC spectra. To reduce the experiment time even further, the ^1^H-^13^C ASAP-HSQC spectra were acquired in combination with nonuniform sampling (NUS). Figure 6 shows a 2D ^1^H-^13^C ASAP-HSQC spectrum of a walnut sample extracted with acetonitrile-*d*_3_/methanol-*d*_4_ (method B).

**Figure 7:**
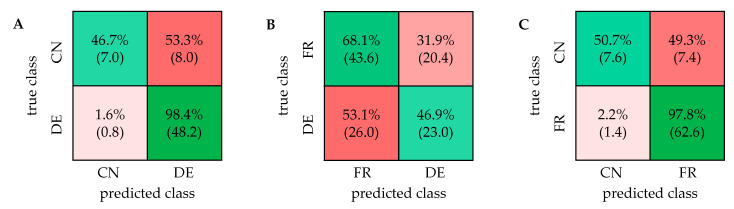
Confusion matrices of all two-class models based on the analysis of the ASAP-HSQC spectra using a linear support vector machine algorithm. CN: China; DE: Germany; FR: France. Accuracies: (**A**) 86.3% (±0.6%); (**B**) 58.9% (±1.3%); (**C**) 88.9% (±1.2%).

The ASAP-HSQC spectrum shows separation of several signals, that occur overlapped in both types of 1D NMR spectra. Especially in the aliphatic region, showing signals of fatty acids, and in the carbohydrate region, signals that occur at the same ^1^H chemical shift are spread across the additional ^13^C frequency domain. In the aromatic region of the spectrum, only few signals can be detected within a total acquisition time of 33 min. In the supplementary information (Figure S5) an ASAP-HSQC spectrum with an increased number of scans (256) is shown, which allows the detection of signals in the aromatic region. However, the total acquisition time of more than four hours acquiring 256 scans is not suitable for high throughput metabolomics studies, which is why spectra, as shown in Figure 6, were acquired for all walnut samples and used for multivariate analysis. PCA and classification were performed similarly as explained above, using the integrated 2D peak intensities as input. The buckets for the PCA were manually defined for all detected signals except the solvent signals and the reference. In total, 40 buckets were defined for further analysis (supplementary information S3). Figure 7 shows all two-class confusion matrices based on the ASAP-HSQC spectra.

In general, the accuracies of the classification using the ASAP-HSQC spectra are partially slightly lower compared to the accuracies obtained by the PSYCHE or ^1^H NOESY spectra. The differentiation of German and Chinese walnut samples yields a high accuracy of 86.3% (±0.6%), as well as for the differentiation of French and Chinese samples with 88.9% (±1.2%). Both confusion matrices in Figure 7A,C show that the accuracy of the classification of the German and French samples each (98.4% for DE; 97.8% for FR) is much higher than the accuracy of the classification of Chinese samples (46.7% for DE/CN; 50.7% for FR/CN). These results seem to be caused by the unequal sample distribution. The classification of French and German samples only yields an accuracy of 58.9% (±1.3%). Due to the close geographical location of Germany and France, the walnut samples show two strongly overlapping clusters in the PCA (supplementary information, Figure S3), already indicating a complicated classification. Especially the samples originating from southern Germany (from Baden-Württemberg and partially from Hesse) are misclassified which can be attributed to the great proximity to France. In Table 1, all calculated accuracies of the classification of the different two-class models after repeated nested CV based on all applied NMR experiments are summarized. The confusion matrices as well as the PCA score plots obtained using three-class models are shown in the supplementary information (Figure S4).

Qualitatively, the score and loading plots of all types of NMR experiments (Figure 2 and supplementary information Figures S2 and S3) show the same trend for the differentiation of each two-class model and lead to comparable results. In all score plots, the German samples (black dots) and the Chinese samples (red dots) show two mostly separated clusters along PC1. The corresponding loading plots each as well show a distribution of the buckets along PC1. Especially the loading plot of the PSYCHE spectra show a clear separation of buckets originating from signals of carbohydrates and those originating from fatty acid signals. Using PSYCHE spectra, the distribution of buckets of carbohydrates and fatty acids indicate that the concentrations of fatty acids and carbohydrates seem to differ between German and Chinese walnut samples. In general, the Chinese walnut samples seem to have slightly higher concentrations of fatty acids, which are mainly linoleic, oleic, α-linolenic, palmitic, and stearic acid [27], while German samples exhibit rather higher concentrations of carbohydrates. Furthermore, in the loading plots of the PSYCHE and the ASAP-HSQC spectra (Figure 2B,C) a clustering of the buckets originating from aromatic compounds is visible. In the loading plot of the 1D ^1^H NOESY spectra (Figure 2A) this clear separation of buckets is less evident since some buckets in the carbohydrate region and most of the buckets in the aliphatic region showing mostly signals of fatty acids overlap. However, classification using the 1D ^1^H NOESY spectra yields slightly higher accuracies, sensitivities, and specificities for almost all models presented. These results are particularly evident in the confusion matrices of the three-class models (Figure S4). Due to the inherently lower signal-to-noise ratio using the PSYCHE or ASAP-HSQC spectra, which additionally leads to a reduced number of buckets that can be used for the classification, and thus due to this loss of information an increase in misclassifications was expected. Nevertheless, the misclassifications increase, especially where they are also high for the 1D NOESY model and are thus in accordance with the trends already observed.

### 2.3. The Potential of 2D NMR Experiments for Determination of the Geographical Origin of Foods

**Figure 8:**
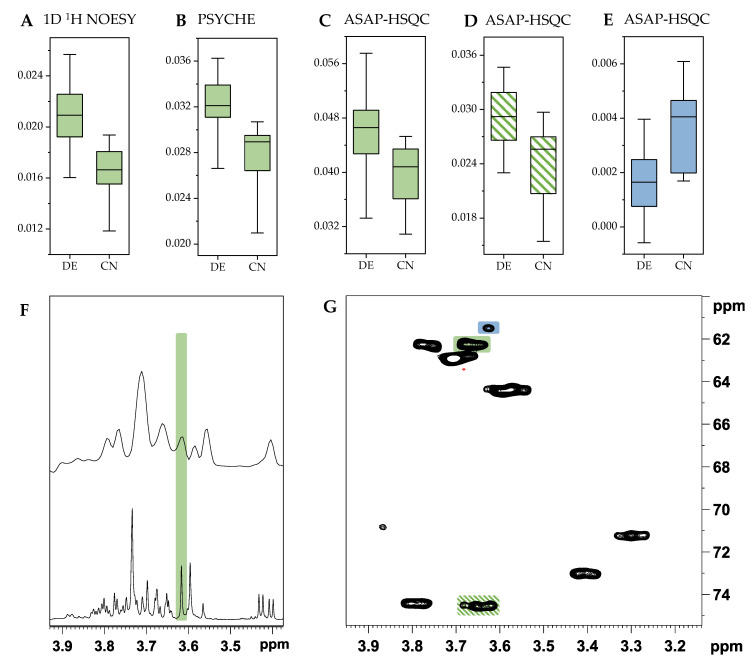
(**A**) Box-whisker plot showing the centered and scaled values of the bucket at 3.61 ppm in the 1D ^1^H NOESY spectra from German (DE) and Chinese (CN) walnut samples. (**B**) Box-whisker plot of the same bucket (3.62 ppm) in the PSYCHE spectra from German and Chinese samples. (**C**) Box-whisker plot of one bucket of sucrose at 3.66/62.2 ppm in the ASAP-HSQC spectra from German and Chinese samples (highlighted green). (**D**) Box-whisker plot of another bucket of sucrose at 3.64/74.5 ppm in the ASAP-HSQC spectra (highlighted green hatched). (**E**) Box-whisker plot of the bucket at 3.63/61.4 ppm in the ASAP-HSQC spectra (highlighted blue). (**F**) Relevant region of the PSYCHE NMR spectrum (upper part) and 1D ^1^H NOESY spectrum (lower part) with the bucket used for the box-whisker plots highlighted in green. (**G**) Carbohydrate region of the ASAP-HSQC spectra with the buckets used for the box-whisker plot highlighted.

Due to the additional frequency domain of 2D ASAP-HSQC spectra some signals show less overlap compared to 1D NMR spectra, as shown above. Especially the detection of low abundant metabolites showing signals in strongly overlapping regions is hindered using 1D NMR spectra. This is shown as an exemplary case in Figure 8.

In Figure 8A–E the box-whisker plots of buckets in the same region of the ^1^H frequency domain in the NMR spectra for the 1D ^1^H NOESY, PSYCHE and ASAP-HSQC spectra each are presented. The selected buckets are highlighted in the respective NMR spectra below (Figure 8F: PSYCHE (upper part) and 1D ^1^H NOESY spectrum (lower part), Figure 8G: ASAP-HSQC spectrum). The box-whisker plot of the bucket at 3.61 ppm in the 1D ^1^H NOESY spectra and at 3.62 ppm in the PSYCHE spectra show mostly the same trend, having higher values for German samples and lower values for Chinese samples. As can be seen from the spectra (Figure 8F) this bucket most likely shows sucrose. Figure 8C,D show the box-whisker plots of two buckets of sucrose in the ASAP-HSQC spectra that occur at the same chemical shift (signal at 3.66/62.2 ppm (highlighted green) and signal at 3.64/74.5 ppm (highlighted green hatched)). As expected, the box-whisker plots of those signals show the same trend as for the one-dimensional NMR spectra since all signals most likely originate from the same metabolite (sucrose). Figure 8E shows the box-whisker plot of the bucket at 3.63/61.4 ppm in the ASAP-HSQC spectrum which does not belong to the signals of sucrose but is in the same chemical shift region of the ^1^H domain as the signals shown before. The associated signal used for the box-whisker plot is highlighted blue in the ASAP-HSQC spectrum in Figure 8G. The trend of the corresponding box-whisker plot differs strongly from the ones shown before having lower values for German and higher values for Chinese walnut samples. This signal at 3.63/61.4 ppm can therefore only be made visible using a 2D HSQC NMR spectrum. In both types of 1D NMR spectra (Figure 8F) the signal of this specific metabolite is strongly overlapped by the high abundant metabolite sucrose, thus impeding the detection of metabolites showing signals in the same chemical shift region. The use of 1D NMR experiments in this particular case thus leads to a loss of information due to compensation of peak intensities, which can lead to a change in the ratio of specific buckets. This example emphasizes the benefits using 2D HSQC spectra for metabolomics studies. Especially low abundant metabolites that might have an impact on the differentiation of sample groups occur less overlapped by high abundant metabolites. Furthermore, this can lead to improved detection and identification of chemical markers that are relevant for differentiation of sample groups.

## 3. Materials and Methods

### 3.1. Reagents and Chemicals

Deuterated solvent methanol-*d*_4_ (99.8%) was purchased from EURISOTOP (Saint-Aubin Cedex, France). Acetonitrile-*d*_3_ (99.5%) was purchased from DEUTERO (Kastellaun, Germany). Octamethylcyclotetrasiloxane (98.0%) was purchased from Alfa Aesar (Ward Hill, Havervill, MA, USA).

### 3.2. Walnut Samples

In total, 128 walnut samples of different geographical origin, varieties, and harvest years 2016 (1), 2017 (30), 2018 (42) and 2019 (55) were analyzed. The sample distribution, including walnut samples from France (64), Germany (49), and China (15), is shown in the supplementary information (Table S11). Most samples were provided by importers and distributors and our collaborators declared the authenticity of the samples.

### 3.3. Sample Preparation

Walnut samples were handled in accordance with previous studies [32]. Freshly delivered walnut samples were dried prior to sample preparation. The walnut samples were shock frozen in liquid nitrogen, and the shell was removed. After another shock freezing process, 100 g of each sample were ground with the addition of 150 g dry ice using a Grindomix GM 300 knife mill equipped with a stainless-steel grinding container and a full metal knife (Retsch, Haan, Germany). The ground walnut samples were then freeze-dried for 48 h and stored at −20 °C afterwards.

### 3.4. Extraction

Extraction method A: For the methanol/acetonitrile extract 302.5 mg (±2.5 mg) lyophilizate was mixed with 500 μL methanol-*d*_4_ and 500 μL acetonitrile-*d*_3_ (+0.5 mM OMS) and two steel balls (Ø = 2 mm) were added. The mixture was extracted in a ball mill for three minutes at 3.1 m/s and it was centrifuged at 14.000 rcf (4 °C) for ten minutes. Then, 350 μL of the supernatant was diluted with 350 μL methanol-*d*_4_. Finally, 600 μL of the diluted extract were transferred to a 5 mm NMR tube.

Extraction method B: For the acquisition of ASAP-HSQC and PSYCHE NMR spectra, the extraction method A was slightly modified in order to achieve higher concentrations of the metabolites since the signal intensities in both types of spectra are generally much lower compared to standard ^1^H NMR spectra. From each walnut sample, 302.5 mg (±2.5 mg) lyophilizate was mixed with 500 µL methanol-*d*_4_ and 500 µL acetonitrile-*d*_3_ (+2.5 mM OMS). After the addition of two steel balls (Ø = 2 mm), the mixture was extracted in a ball mill for three minutes at 3.1 m/s and centrifuged at 14 000 rcf and 4 °C for ten minutes afterwards. Then, 600 µL of the supernatant were mixed with 100 µL of methanol-*d*_4_ and 600 µL of this extract solution were transferred into a 5 mm NMR tube.

### 3.5. NMR Acquisition

All NMR spectra were acquired on a Bruker Avance III HD 400 MHz NMR spectrometer using TopSpin 3.6.2 (Bruker Biospin GmbH, Rheinstetten, Germany) equipped with a 5 mm BBO probe and operating at 400.13 MHz and 300 K.

The noesygppr1d pulse sequence was used for acquisition of water suppressed ^1^H NMR spectra applying the digitization mode baseopt. For every sample, spectra were recorded with number of scans (NS) of 128, 65,536 complex data points and a spectral width of 8417.5 Hz. The RG was set to 64 and the transmitter frequency offset to 1778.2 Hz. The total duration time was 17 min.

The ASAP-HSQC spectra were acquired with the pulse sequence from Schulze-Sünninghausen et al. [22]. In order to combine the ASAP-HSQC experiment with nonuniform-sampling, the syntax of the mc-command was changed. All spectra were recorded with 32 scans and 128 dummy scans, 1024 data points were recorded in F2 as well as 512 increments in F1. The spectral width in F2 was 11 ppm and 165 ppm in F1. The relaxation delay was set to 0.3 s. In addition, nonuniform sampling was used so that only 25% of the increments were actually recorded. The mean coupling constant ^1^*J*_CH_ for the INEPT transfer was set to 145 Hz, resulting in a delay Δ of 1.72 ms for the inverse INEPT transfer. A value of 210 Hz was selected for CNST3, resulting in a delay Δ′ of 1.19 ms. The mixing time for the DIPSI-2 sequence was 30 ms. A garp4 sequence was used for the ^13^C decoupling during FID recording. The total duration of the experiment was 33 min.

The homonuclear broadband decoupled PSYCHE NMR spectra were acquired with the reset-psyche pulse sequence from Bruker Biospin GmbH, which relies on the psyche pulse sequence from Foroozandeh et al. [14,18,19]. The spectra were recorded with 128 scans, two previous dummy scans, 16,384 data points in the direct dimension and 20 data chunks. The spectral width was set to 80 Hz in F1 and 8000 Hz in F2. The relaxation delay was 0.5 s. A flip angle *β* for the CHIRP pulse element of 45° was chosen. The number of complex points of each FID block κ was set to 64, resulting in a block length of 8 ms. The total duration of the experiment was 71 min.

### 3.6. NMR Data Processing and Analysis

For the 1D ^1^H NOESY spectra the FIDs were weighed by an exponential function with a line broadening factor of 0.3 Hz and Fourier transformed. The spectra obtained were calibrated to the OMS signal (*δ* = 0.085 ppm) and processed by automatic phase and baseline correction with Topspin 3.5 (Bruker Biospin, Rheinstetten, Germany).

The ASAP-HSQC spectra that were acquired in combination with NUS were processed using the Compressed Sensing (CS) Algorithm with Iterative Soft Thresholding (IST) in TopSpin 4.0.8 (Bruker Biospin GmbH, Rheinstetten, Germany). Zero filling up to 2048 data points was applied in the direct and indirect dimensions. In addition, complex linear forward prediction was performed in the F1 dimension with 32 coefficients. A Hilbert transformation was performed in the F2 dimension to calculate the imaginary part of the FID. Afterwards, a manual phase correction, as well as an automatic baseline correction with a first-degree polynomial function in both dimensions, was performed. The spectra were calibrated to the OMS signal (*δ* (^1^H) = 0.085 ppm, *δ* (^13^C) = 0.883 ppm).

The PSYCHE spectra were processed with the AU program proc_reset in TopSpin 4.0.8 (Bruker Biospin, Rheinstetten, Germany), whereby the number of columns for averaging was set to 1. In the resulting one-dimensional pure shift spectra, zero filling was applied to 4096 data points. In addition, a window multiplication with a QSINE function with sine bell shift of 2 was performed, and the spectra were calibrated to the OMS signal (*δ* = 0.085 ppm). Furthermore, a manual phase correction and an automatic baseline correction with a first-degree polynomial function were performed.

### 3.7. Multivariate Analysis

The software Amix (Version 3.9.15, Bruker Biospin, Rheinstetten, Germany) was used for analyzing the spectra. For ^1^H NOESY, PSYCHE, and ASAP-HSQC spectra, the buckets were defined manually with variable size, since the signal pattern is different in each spectrum and as this led to the best results regarding PCA (number of buckets: 183 for 1D ^1^H NOESY, 30 for PSYCHE and 40 for ASAP-HSQC spectra). The selected buckets (supplementary information S1–S3) were scaled to total intensity (rows). The PCA was carried out using the data obtained. The scaling of bucket variables (columns) is based on unit variance and the default confidence level was set to 95%. The number of principal components was set to a minimum explained variance of 95%. The Kruskal-Wallis significance analysis of variables based on a modified procedure of Goodpaster et al. was performed with a confidence level set to 95% [36]. The *p*-value was Bonferroni-corrected, and buckets were indicated as significant if *p* < 0.00027322 for the 1D ^1^H NOESY spectra, if *p* < 0.0016667 for the PSYCHE spectra or if *p* < 0.00125 for the ASAP-HSQC spectra. The buckets with their corresponding *p*-values for each differentiation are shown in the supplementary information (Tables S1–S9). The PCA score and loading plots were plotted using the software Origin 2019 (OriginLab, Northampton, MA, USA). For building classification models, the software Matlab R2019a/b (The Mathworks, Inc., Natick, MA, USA) including the Classification Learner App was used. A total of three binary classification models for every kind of NMR spectra were tested. The normalized bucket table was exported from Amix to Matlab. Afterwards, a repeated nested CV was performed [33,35]. First, the sample set (e.g., CN/DE) was randomly separated into five equal parts. In each part, the samples were stratified by origin. Four of these parts were combined to create a training set with which a model was obtained by training the linear support vector machine classifier in combination with an internal five-fold CV. In the internal five-fold CV, which is implemented in the software Matlab, the division into training and test sets was conducted randomized. The validation of the model obtained was performed by using the one part that was left out (test set). The process was repeated five times (five-fold outer CV) to assess the model performance [33] and thus, each part was used as a test set once. In total, the process was repeated five times with randomly remixed parts. The accuracies of the five models for a classification obtained by the repeated nested CV were averaged and given with the corresponding deviation. For the classification based on the different types of NMR spectra, the same composition of training and test sets each was used. The final result of a model was presented as mean in a confusion matrix. For classification, the support vector machine classifier with a linear kernel function and the box constraint level set to 1 was used, since this led to the best results regarding the accuracy of the models.

## 4. Conclusions

This study demonstrates the utility of different NMR experiments (1D ^1^H NOESY, PSYCHE, and ^1^H-^13^C ASAP-HSQC) regarding the classification of walnut samples of different geographical origin (France, China, and Germany) in an untargeted metabolomics approach using the mid-polar acetonitrile/methanol (1:1) extract. Multivariate analysis such as PCA and classification using linear SVM showed that the highest classification accuracies can be obtained by using 1D ^1^H NOESY spectra. Compared to previous studies, it was shown that the accuracies of the classification models based on two different extraction methods were similar [32]. Similarities were also found in the incorrectly classified samples. Furthermore, the total acquisition time of 1D NOESY spectra is lowest (~17 min) in comparison to the other types of spectra. High classification accuracies can also be obtained using homonuclear decoupled ^1^H NMR spectra (PSYCHE) but disadvantages occur regarding the overall acquisition time of PSYCHE spectra (~71 min) and the complicated processing technique, which can additionally lead to the occurrence of artefacts in the spectra. Additionally, classification accuracies decrease slightly using the PSYCHE or ASAP-HSQC spectra, which can be expected from the loss in signal-to-noise. However, the use of 2D ^1^H-^13^C HSQC spectra for subsequent multivariate analysis is an advantageous method since clearer separation of the signals is possible due to the additional ^13^C frequency domain. This also contributes to a greater distinction of the buckets used for classification since they do not overlap with other high abundant metabolites, thus reducing the risk of compensation of bucket intensities, which could lead to a loss of information. Furthermore, the use of 2D ^1^H-^13^C HSQC spectra may simplify the identification of the metabolites at the same time since information about the ^13^C chemical shifts can be obtained simultaneously. In general, reliable and robust models were obtained for the classification of walnut samples from Germany, France, and China. In future studies, data fusion will be conducted to improve the accuracies of the models. Another focus will be the use of 2D ^1^H-^13^C HSQC experiments for simple identification of metabolites from walnut.

## Figures and Tables

**Table 1:** Accuracies of the classification of all three two-class models (DE/CN, FR/DE, FR/CN) based on the different NMR experiments (^1^H NOESY, PSYCHE, ASAP-HSQC). CN: China; DE: Germany; FR: France.

Model	1D ^1^H NOESY	PSYCHE	ASAP-HSQC
DE/CN	95.9% (±0.8%)	90.9% (±1.5%)	86.3% (±0.6%)
FR/DE	83.4% (±2.0%)	68.3% (±2.8%)	58.9% (±1.3%)
FR/CN	93.7% (±1.1%)	88.9% (±1.2%)	88.9% (±1.2%)

## Data Availability

The data presented in this study are openly available in MetaboLights at doi:10.1093/nar/gkz1019, reference number MTBLS2341 [37].

## Supplementary Materials

The following are available online at https://www.mdpi.com/2218-1989/11/1/39/s1, Tables S1–S9: Buckets with corresponding *p*-values for each differentiation (DE/CN; FR/DE; FR/CN) using 1D ^1^H NOESY, PSYCHE and ASAP-HSQC spectra. Table S10: Comparison of the accuracies of classification models based on the mid-polar extract and the polar extraction method from previous studies [32]. Table S11: Information about walnut samples. S1–S3: List of variable sized buckets used for building the classification models. Figure S1: ^1^H NOESY spectra of the stability measurement. Figures S2 and S3: PCA score and loading plot of the differentiation of walnut samples of two countries each (FR/CN; FR/DE). Figure S4: Confusion matrices and PCA score plots using three-class models. Figure S5: ASAP-HSQC spectrum of a walnut extract acquired with 256 scans.

## Sample Availability

Walnut samples are not available from the authors.

## References

[1] Esslinger S., Riedl J., Fauhl-Hassek C. (2014). Potential and limitations of non-targeted fingerprinting for authentication of food in official control. Food Res. Int..

[2] Laghi L., Picone G., Capozzi F. (2014). Nuclear magnetic resonance for foodomics beyond food analysis. TrAC-Trends Anal. Chem..

[3] Bachmann R., Klockmann S., Haerdter J., Fischer M., Hackl T. (2018). 1H NMR Spectroscopy for Determination of the Geographical Origin of Hazelnuts. J. Agric. Food Chem..

[4] Sharma R., Gogna N., Singh H., Dorai K. (2017). Fast profiling of metabolite mixtures using chemometric analysis of a speeded-up 2D heteronuclear correlation NMR experiment. RSC Adv..

[5] Mozumder N.H.M.R., Lee Y.R., Hwang K.H., Lee M.S., Kim E.H., Hong Y.S. (2020). Characterization of tea leaf metabolites dependent on tea (Camellia sinensis) plant age through 1H NMR-based metabolomics. Appl. Biol. Chem..

[6] Wei D., Chen D., Lou Y., Ye Y., Yang R. (2016). Metabolomic profile characteristics of pyropia haitanensis as affected by harvest time. Food Sci. Technol. Res..

[7] Schmitt C., Bastek T., Stelzer A., Schneider T., Fischer M., Hackl T. (2020). Detection of Peanut Adulteration in Food Samples by Nuclear Magnetic Resonance Spectroscopy. J. Agric. Food Chem..

[8] Emwas A.H., Roy R., McKay R.T., Tenori L., Saccenti E., Gowda G.A.N., Raftery D., Alahmari F., Jaremko L., Jaremko M. (2019). NMR spectroscopy for metabolomics research. Metabolites.

[9] Wishart D.S. (2008). Metabolomics: Applications to food science and nutrition research. Trends Food Sci. Technol..

[10] Pan Z., Raftery D. (2007). Comparing and combining NMR spectroscopy and mass spectrometry in metabolomics. Anal. Bioanal. Chem..

[11] Cubero-Leon E., Peñalver R., Maquet A. (2014). Review on metabolomics for food authentication. Food Res. Int..

[12] Markley J.L., Brüschweiler R., Edison A.S., Eghbalnia H.R., Powers R., Raftery D., Wishart D.S. (2018). The Future of NMR-Based Metabolomics. Curr. Opin. Biotechnol..

[13] Zangger K., Sterk H. (1997). Homonuclear Broadband-Decoupled NMR Spectra. J. Magn. Reson..

[14] Foroozandeh M., Morris G.A., Nilsson M. (2018). Psyche Pure Shift NMR Spectroscopy. Chem. Eur. J..

[15] Aue W.P., Karhan J., Ernst R.R. (1976). Homonuclear broad band decoupling and two-dimensional J-resolved NMR spectroscopy. J. Chem. Phys..

[16] Zangger K. (2015). Pure shift NMR. Prog. Nucl. Magn. Reson. Spectrosc..

[17] Bo Y., Feng J., Xu J.J., Huang Y., Cai H., Cui X., Dong J., Ding S., Chen Z. (2019). High-resolution pure shift NMR spectroscopy offers better metabolite discrimination in food quality analysis. Food Res. Int..

[18] Foroozandeh M., Adams R.W., Meharry N.J., Jeannerat D., Nilsson M., Morris G.A. (2014). Ultrahigh-resolution NMR spectroscopy. Angew. Chem. Int. Ed..

[19] Castañar L., Parella T. (2015). Broadband 1H homodecoupled NMR experiments: Recent developments, methods and applications. Magn. Reson. Chem..

[20] Santacruz L., Hurtado D.X., Doohan R., Thomas O.P., Puyana M., Tello E. (2020). Metabolomic study of soft corals from the Colombian Caribbean: PSYCHE and ^1^H-NMR comparative analysis. Sci. Rep..

[21] Lopez J.M., Cabrera R., Maruenda H. (2019). Ultra-Clean Pure Shift 1H-NMR applied to metabolomics profiling. Sci. Rep..

[22] Schulze-Sünninghausen D., Becker J., Luy B. (2014). Rapid Heteronuclear Single Quantum Correlation NMR Spectra at Natural Abundance. J. Am. Chem. Soc..

[23] Kupce E., Freeman R. (2007). Fast multidimensional NMR by polarization sharing. Magn. Reson. Chem..

[24] Schulze-Sünninghausen D., Becker J., Koos M.R.M., Luy B. (2017). Improvements, extensions, and practical aspects of rapid ASAP-HSQC and ALSOFAST-HSQC pulse sequences for studying small molecules at natural abundance. J. Magn. Reson..

[25] Le Guennec A., Giraudeau P., Caldarelli S. (2014). Evaluation of fast 2D NMR for metabolomics. Anal. Chem..

[26] Puig-Castellví F., Pérez Y., Piña B., Tauler R., Alfonso I. (2018). Comparative analysis of 1H NMR and 1H-13C HSQC NMR metabolomics to understand the effects of medium composition in yeast growth. Anal. Chem..

[27] Popescu R., Ionete R.E., Botoran O.R., Costinel D., Bucura F., Geana E.I., Alabedallat Y.F.J., Botu M. (2019). ^1^H NMR profiling and carbon isotope discrimination as tools for the comparative assessment of walnut (*Juglans regia* L.) cultivars with various geographical and genetic origins—A preliminary study. Molecules.

[28] Li B., Wang H., Zhao Q., Ouyang J., Wu Y. (2015). Rapid detection of authenticity and adulteration of walnut oil by FTIR and fluorescence spectroscopy: A comparative study. Food Chem..

[29] Esteki M., Farajmand B., Amanifar S., Barkhordari R., Ahadiyan Z., Dashtaki E., Mohammadlou M., Heyden Y.V. (2017). Classification and authentication of Iranian walnuts according to their geographical origin based on gas chromatographic fatty acid fingerprint analysis using pattern recognition methods. Chemom. Intell. Lab. Syst..

[30] Beckonert O., Keun H.C., Ebbels T.M.D., Bundy J., Holmes E., Lindon J.C., Nicholson J.K. (2007). Metabolic profiling, metabolomic and metabonomic procedures for NMR spectroscopy of urine, plasma, serum and tissue extracts. Nat. Protoc..

[31] McKay R.T. (2011). How the 1D-NOESY Suppresses Solvent Signal in Metabonomics NMR Spectroscopy: An Examination of the Pulse Sequence Components and Evolution. Concepts Magn. Reson. Part A.

[32] Schmitt C., Schneider T., Rumask L., Fischer M., Hackl T. (2020). Food Profiling: Determination of the Geographical Origin of Walnuts by ^1^H NMR Spectroscopy Using the Polar Extract. J. Agric. Food Chem..

[33] Varma S., Simon R. (2006). Bias in error estimation when using cross-validation for model selection. BMC Bioinform..

[34] Westerhuis J.A., Hoefsloot H.C.J., Smit S., Vis D.J., Smilde A.K., Velzen E.J.J., Duijnhoven J.P.M., Dorsten F.A. (2008). Assessment of PLSDA cross validation. Metabolomics.

[35] Krstajic D., Buturovic L.J., Leahy D.E., Thomas S. (2014). Cross-validation pitfalls when selecting and assessing regression and classification models. J. Cheminform..

[36] Goodpaster A.M., Romick-Rosendale L.E., Kennedy M.A. (2010). Statistical significance analysis of nuclear magnetic resonance-based metabonomics data. Anal. Biochem..

[37] Haug K., Cochrane K., Nainala V.C., Williams M., Chang J., Jayaseelan K.V., O’Donovan C. (2020). MetaboLights: A resource evolving in response to the needs of its scientific community. Nuc. Acids Res..

