# Supplementary material for: Comparison of Regular, Pure Shift, and Fast 2D NMR Experiments for Determination of the Geographical Origin of Walnuts
Source: Metabolites. 2021 Jan 8;11(1):39. doi: 10.3390/metabo11010039 (PMC7827277; doi:10.3390/metabo11010039)
Supplement: Supplementary file 1 [file metabolites-11-00039-s001.pdf]

Supplementary Materials for

Comparison of Regular, Pure Shift, and Fast 2D NMR Experiments for Determination of the Geographical Origin of Walnuts

Stephanie Watermann [1], Caroline Schmitt [1], Tobias Schneider [1] and Thomas Hackl [1,2,] ×

[1] Institute of Organic Chemistry, University of Hamburg, Martin-Luther-King-Platz 6, 20146 Hamburg, Germany; stephanie.watermann@chemie.uni-hamburg.de (S.W.); caroline.schmitt@chemie.uni-hamburg.de (C.S.); tobias.schneider@chemie.uni-hamburg.de (T.S.)

[2] Hamburg School of Food Science – Institute of Food Chemistry, University of Hamburg, Grindelallee 117, 20146 Hamburg, Germany

× Correspondence: thomas.hackl@chemie.uni-hamburg.de; Tel.: +49-40-42838-2804 (T.H.)

Supporting Information – Content

Buckets with corresponding *p*-values for each differentiation (DE/CN; FR/DE; FR/CN) using 1D ^{1}H NOESY, PSYCHE and ASAP-HSQC spectra (Table S1 - S9) .... S-III

Comparison of the accuracies of classification models based on the mid-polar extract and the polar extraction method from previous studies (Table S10) .... S-X

Information about walnut samples (Table S11) .... S-XI

List of variable sized buckets used for building the classification models (S1–S3) .... S-XVII

^{1}H NOESY spectra of the stability measurement (Figure S1) .... S-XIX

PCA score and loading plot of the differentiation of walnut samples of two countries each (FR/CN; FR/DE) (Figure S2 and S3) .... S-XX

Confusion matrices and PCA score plots of the differentiation of walnut samples using three-class models (Figure S4) .... S-XXII

ASAP-HSQC spectrum of a walnut extract acquired with 256 scans (Figure S5) .... S-XXIII

Table S1. Buckets with corresponding *p*-values for differentiation of samples from Germany and China based on 1D ^{1}H NOESY spectra. Significant buckets (44) with a *p*-value < 0.00027322 are highlighted.

ppm	*p*-value	ppm	*p*-value	ppm	*p*-value	ppm	*p*-value
0.350	6.48×10^{-2}	3.269	4.48×10^{-5}	5.003	2.25×10^{-1}	6.497	7.10×10^{-1}
0.557	6.96×10^{-2}	3.322	1.29×10^{-6}	5.112	2.02×10^{-1}	6.526	6.51×10^{-1}
0.710	3.96×10^{-1}	3.335	7.69×10^{-1}	5.211	1.86×10^{-1}	6.552	2.44×10^{-1}
0.743	9.99×10^{-5}	3.347	9.34×10^{-7}	5.245	1.10×10^{-2}	6.584	6.98×10^{-1}
0.799	3.71×10^{-1}	3.362	2.19×10^{-1}	5.257	3.28×10^{-3}	6.605	5.11×10^{-1}
0.822	7.57×10^{-1}	3.372	9.18×10^{-1}	5.268	2.93×10^{-2}	6.626	4.71×10^{-1}
0.842	1.66×10^{-1}	3.400	2.49×10^{-7}	5.281	9.45×10^{-2}	6.670	2.31×10^{-1}
0.859	1.70×10^{-1}	3.423	1.10×10^{-6}	5.292	1.15×10^{-2}	6.688	2.13×10^{-1}
0.899	1.51×10^{-6}	3.442	8.77×10^{-6}	5.313	3.74×10^{-4}	6.716	4.42×10^{-1}
0.936	1.01×10^{-1}	3.464	3.57×10^{-2}	5.346	1.69×10^{-5}	6.736	5.85×10^{-1}
0.967	6.98×10^{-1}	3.483	6.48×10^{-2}	5.380	9.66×10^{-8}	6.760	5.01×10^{-1}
1.001	8.32×10^{-3}	3.496	8.93×10^{-1}	5.402	7.32×10^{-7}	6.781	3.79×10^{-1}
1.020	5.15×10^{-3}	3.510	9.18×10^{-1}	5.417	7.21×10^{-1}	6.832	7.21×10^{-1}
1.034	1.51×10^{-6}	3.532	3.38×10^{-1}	5.439	6.06×10^{-1}	6.886	4.05×10^{-1}
1.052	1.77×10^{-7}	3.559	4.14×10^{-6}	5.463	7.21×10^{-1}	6.900	1.70×10^{-1}
1.100	6.26×10^{-2}	3.589	2.24×10^{-5}	5.486	5.42×10^{-1}	6.918	8.84×10^{-2}
1.115	1.66×10^{-3}	3.612	5.27×10^{-7}	5.531	6.86×10^{-1}	6.934	2.20×10^{-2}
1.146	1.15×10^{-1}	3.654	3.05×10^{-6}	5.585	5.82×10^{-2}	6.968	6.51×10^{-1}
1.163	1.05×10^{-2}	3.730	3.56×10^{-6}	5.623	3.88×10^{-1}	6.991	3.57×10^{-2}
1.181	6.01×10^{-4}	3.806	6.22×10^{-7}	5.669	3.62×10^{-1}	7.002	2.31×10^{-1}
1.219	4.05×10^{-1}	3.841	3.11×10^{-4}	5.686	1.70×10^{-1}	7.010	2.64×10^{-1}
1.325	4.82×10^{-6}	3.855	2.15×10^{-4}	5.696	1.91×10^{-1}	7.035	8.84×10^{-2}
1.430	1.19×10^{-6}	3.877	6.75×10^{-7}	5.707	1.57×10^{-2}	7.057	1.15×10^{-1}
1.449	8.14×10^{-6}	3.944	6.29×10^{-1}	5.718	3.44×10^{-2}	7.077	9.18×10^{-1}
1.594	1.02×10^{-5}	3.970	1.78×10^{-4}	5.761	3.18×10^{-2}	7.100	2.38×10^{-1}
1.816	2.77×10^{-5}	4.004	5.27×10^{-7}	5.785	3.07×10^{-1}	7.112	8.68×10^{-1}
1.895	2.49×10^{-2}	4.038	4.47×10^{-7}	5.804	1.86×10^{-2}	7.118	7.57×10^{-1}
1.928	8.02×10^{-4}	4.059	5.42×10^{-2}	5.824	8.68×10^{-1}	7.138	7.82×10^{-1}
1.956	7.15×10^{-4}	4.082	2.42×10^{-6}	5.840	2.99×10^{-1}	7.194	1.91×10^{-1}
2.007	1.78×10^{-4}	4.097	2.61×10^{-6}	5.916	3.14×10^{-1}	7.205	3.07×10^{-1}
2.035	1.19×10^{-6}	4.107	3.05×10^{-6}	6.047	8.84×10^{-2}	7.227	2.57×10^{-1}
2.053	3.29×10^{-6}	4.122	3.91×10^{-5}	6.074	7.33×10^{-1}	7.283	7.33×10^{-1}
2.070	7.19×10^{-5}	4.132	1.77×10^{-6}	6.102	6.18×10^{-1}	7.299	8.84×10^{-2}
2.088	8.72×10^{-3}	4.148	1.77×10^{-6}	6.117	7.21×10^{-1}	7.318	5.68×10^{-3}
2.104	1.66×10^{-1}	4.161	1.36×10^{-5}	6.129	6.96×10^{-2}	7.401	4.81×10^{-1}
2.119	3.41×10^{-5}	4.168	2.25×10^{-1}	6.141	7.94×10^{-1}	7.508	9.81×10^{-1}
2.154	1.69×10^{-5}	4.178	5.88×10^{-5}	6.161	2.31×10^{-1}	7.595	2.49×10^{-2}
2.491	8.93×10^{-1}	4.223	9.56×10^{-3}	6.193	1.86×10^{-1}	7.648	4.33×10^{-2}

2.739	1.20×10^{-2}	4.562	9.18×10^{-1}	6.228	7.45×10^{-1}	7.667	2.64×10^{-1}
2.796	9.56×10^{-3}	4.599	3.22×10^{-1}	6.249	2.78×10^{-1}	7.755	6.51×10^{-1}
2.852	8.18×10^{-1}	4.679	6.63×10^{-1}	6.299	7.45×10^{-1}	7.959	9.31×10^{-1}
2.883	1.86×10^{-2}	4.808	6.40×10^{-1}	6.346	3.71×10^{-1}	8.021	2.11×10^{-2}
2.914	6.26×10^{-2}	4.827	3.88×10^{-1}	6.386	9.81×10^{-1}	8.081	7.94×10^{-1}
2.951	1.38×10^{-1}	4.849	1.66×10^{-3}	6.410	3.14×10^{-1}	8.197	5.11×10^{-1}
3.022	6.04×10^{-2}	4.872	1.49×10^{-7}	6.438	1.70×10^{-1}	8.478	9.81×10^{-1}
3.233	1.47×10^{-1}	4.930	3.22×10^{-1}	6.461	6.86×10^{-1}		

Table S2. Buckets with corresponding *p*-values for differentiation of samples from Germany and France based on 1D ^{1}H NOESY spectra. Significant buckets (9) with a *p*-value < 0.00027322 are highlighted.

ppm	*p*-value	ppm	*p*-value	ppm	*p*-value	ppm	*p*-value
0.350	8.53×10^{-1}	3.269	9.33×10^{-6}	5.003	2.07×10^{-1}	6.497	7.46×10^{-1}
0.557	6.77×10^{-1}	3.322	7.07×10^{-2}	5.112	2.63×10^{-1}	6.526	3.30×10^{-1}
0.710	3.88×10^{-1}	3.335	5.98×10^{-1}	5.211	1.40×10^{-1}	6.552	1.81×10^{-1}
0.743	1.20×10^{-1}	3.347	4.63×10^{-2}	5.245	5.52×10^{-2}	6.584	4.98×10^{-1}
0.799	6.31×10^{-1}	3.362	1.51×10^{-1}	5.257	4.89×10^{-2}	6.605	8.17×10^{-1}
0.822	9.82×10^{-1}	3.372	9.35×10^{-1}	5.268	2.61×10^{-1}	6.626	2.13×10^{-1}
0.842	6.43×10^{-1}	3.400	3.86×10^{-2}	5.281	2.86×10^{-1}	6.670	2.24×10^{-2}
0.859	3.02×10^{-1}	3.423	6.13×10^{-2}	5.292	3.57×10^{-1}	6.688	8.12×10^{-2}
0.899	1.49×10^{-1}	3.442	2.09×10^{-4}	5.313	2.49×10^{-1}	6.716	3.42×10^{-1}
0.936	8.94×10^{-1}	3.464	2.73×10^{-2}	5.346	4.62×10^{-1}	6.736	4.20×10^{-2}
0.967	3.39×10^{-1}	3.483	3.70×10^{-3}	5.380	3.07×10^{-2}	6.760	1.99×10^{-2}
1.001	3.80×10^{-5}	3.496	2.11×10^{-1}	5.402	6.54×10^{-2}	6.781	7.54×10^{-1}
1.020	5.02×10^{-1}	3.510	7.31×10^{-3}	5.417	8.57×10^{-1}	6.832	1.49×10^{-1}
1.034	4.69×10^{-3}	3.532	4.34×10^{-1}	5.439	6.14×10^{-1}	6.886	2.24×10^{-1}
1.052	1.36×10^{-5}	3.559	1.72×10^{-1}	5.463	1.90×10^{-1}	6.900	7.46×10^{-1}
1.100	5.94×10^{-1}	3.589	1.48×10^{-1}	5.486	3.94×10^{-1}	6.918	4.94×10^{-1}
1.115	6.43×10^{-1}	3.612	3.81×10^{-2}	5.531	1.49×10^{-1}	6.934	8.39×10^{-1}
1.146	3.28×10^{-1}	3.654	5.23×10^{-2}	5.585	9.03×10^{-1}	6.968	2.11×10^{-1}
1.163	7.90×10^{-1}	3.730	8.53×10^{-2}	5.623	8.39×10^{-1}	6.991	8.35×10^{-1}
1.181	8.48×10^{-1}	3.806	6.89×10^{-2}	5.669	7.72×10^{-1}	7.002	5.16×10^{-1}
1.219	5.02×10^{-1}	3.841	6.58×10^{-4}	5.686	9.91×10^{-1}	7.010	8.48×10^{-1}
1.325	1.23×10^{-1}	3.855	1.70×10^{-2}	5.696	7.41×10^{-1}	7.035	1.00×10^{0}
1.430	2.34×10^{-6}	3.877	1.82×10^{-4}	5.707	6.77×10^{-1}	7.057	3.07×10^{-2}
1.449	1.19×10^{-5}	3.944	6.37×10^{-2}	5.718	4.44×10^{-1}	7.077	8.39×10^{-1}
1.594	1.49×10^{-1}	3.970	7.94×10^{-1}	5.761	8.53×10^{-1}	7.100	4.98×10^{-1}
1.816	4.66×10^{-4}	4.004	7.16×10^{-2}	5.785	9.44×10^{-3}	7.112	6.63×10^{-2}
1.895	2.24×10^{-1}	4.038	6.05×10^{-2}	5.804	9.31×10^{-1}	7.118	5.32×10^{-1}
1.928	1.59×10^{-1}	4.059	1.98×10^{-1}	5.824	8.71×10^{-1}	7.138	4.87×10^{-1}
1.956	3.61×10^{-5}	4.082	4.18×10^{-4}	5.840	5.23×10^{-3}	7.194	4.48×10^{-1}

2.007	2.77×10^{-2}	4.097	7.95×10^{-4}	5.916	2.39×10^{-2}	7.205	7.94×10^{-1}
2.035	6.47×10^{-1}	4.107	3.11×10^{-1}	6.047	6.31×10^{-1}	7.227	7.85×10^{-1}
2.053	2.05×10^{-1}	4.122	3.98×10^{-1}	6.074	3.66×10^{-1}	7.283	2.56×10^{-1}
2.070	1.38×10^{-1}	4.132	2.42×10^{-2}	6.102	2.68×10^{-1}	7.299	2.69×10^{-3}
2.088	2.17×10^{-1}	4.148	1.81×10^{-1}	6.117	2.86×10^{-1}	7.318	7.70×10^{-3}
2.104	4.07×10^{-1}	4.161	6.80×10^{-2}	6.129	4.44×10^{-1}	7.401	4.27×10^{-1}
2.119	4.65×10^{-1}	4.168	2.47×10^{-1}	6.141	7.16×10^{-2}	7.508	2.63×10^{-1}
2.154	2.56×10^{-4}	4.178	2.61×10^{-2}	6.161	3.75×10^{-1}	7.595	9.82×10^{-1}
2.491	2.46×10^{-2}	4.223	5.43×10^{-1}	6.193	2.42×10^{-1}	7.648	2.50×10^{-2}
2.739	2.76×10^{-1}	4.562	3.54×10^{-1}	6.228	1.68×10^{-1}	7.667	4.95×10^{-2}
2.796	2.44×10^{-1}	4.599	1.61×10^{-1}	6.249	1.50×10^{-2}	7.755	7.72×10^{-1}
2.852	4.69×10^{-2}	4.679	6.56×10^{-1}	6.299	4.17×10^{-1}	7.959	5.16×10^{-2}
2.883	1.10×10^{-1}	4.808	8.53×10^{-1}	6.346	3.48×10^{-1}	8.021	9.63×10^{-1}
2.914	1.72×10^{-1}	4.827	8.76×10^{-1}	6.386	5.16×10^{-1}	8.081	2.61×10^{-1}
2.951	4.50×10^{-2}	4.849	7.63×10^{-2}	6.410	8.08×10^{-1}	8.197	9.91×10^{-1}
3.022	8.68×10^{-3}	4.872	3.98×10^{-3}	6.438	7.99×10^{-1}	8.478	6.68×10^{-1}
3.233	6.13×10^{-2}	4.930	6.39×10^{-1}	6.461	1.15×10^{-1}		

Table S3. Buckets with corresponding *p*-values for differentiation of samples from China and France based on 1D ^{1}H NOESY spectra. Significant buckets (30) with a *p*-value < 0.00027322 are highlighted.

ppm	*p*-value	ppm	*p*-value	ppm	*p*-value	ppm	*p*-value
0.350	5.42×10^{-2}	3.269	7.20×10^{-3}	5.003	4.31×10^{-1}	6.497	7.93×10^{-1}
0.557	1.08×10^{-2}	3.322	5.69×10^{-6}	5.112	3.88×10^{-1}	6.526	9.70×10^{-1}
0.710	8.81×10^{-1}	3.335	9.40×10^{-1}	5.211	2.01×10^{-2}	6.552	5.16×10^{-1}
0.743	9.25×10^{-4}	3.347	9.64×10^{-6}	5.245	7.19×10^{-2}	6.584	9.20×10^{-1}
0.799	7.80×10^{-2}	3.362	6.00×10^{-1}	5.257	1.29×10^{-2}	6.605	6.17×10^{-1}
0.822	9.40×10^{-1}	3.372	9.30×10^{-1}	5.268	4.04×10^{-2}	6.626	8.41×10^{-1}
0.842	3.95×10^{-1}	3.400	4.76×10^{-6}	5.281	1.85×10^{-1}	6.670	8.12×10^{-1}
0.859	3.62×10^{-1}	3.423	1.21×10^{-5}	5.292	3.46×10^{-2}	6.688	4.31×10^{-1}
0.899	1.36×10^{-5}	3.442	2.89×10^{-4}	5.313	2.11×10^{-3}	6.716	2.50×10^{-1}
0.936	1.58×10^{-1}	3.464	1.51×10^{-1}	5.346	5.40×10^{-5}	6.736	8.68×10^{-2}
0.967	9.40×10^{-1}	3.483	5.74×10^{-1}	5.380	1.31×10^{-6}	6.760	5.58×10^{-2}
1.001	4.46×10^{-1}	3.496	7.45×10^{-1}	5.402	1.61×10^{-5}	6.781	2.55×10^{-1}
1.020	4.04×10^{-2}	3.510	1.69×10^{-1}	5.417	6.08×10^{-1}	6.832	4.24×10^{-1}
1.034	8.35×10^{-3}	3.532	2.78×10^{-2}	5.439	7.26×10^{-1}	6.886	1.24×10^{-1}
1.052	7.72×10^{-4}	3.559	1.08×10^{-5}	5.463	7.45×10^{-1}	6.900	1.21×10^{-1}
1.100	2.45×10^{-1}	3.589	1.08×10^{-5}	5.486	2.61×10^{-1}	6.918	3.92×10^{-2}
1.115	2.70×10^{-3}	3.612	5.36×10^{-6}	5.531	1.58×10^{-1}	6.934	1.94×10^{-2}
1.146	6.25×10^{-2}	3.654	1.44×10^{-5}	5.585	8.01×10^{-2}	6.968	1.69×10^{-1}
1.163	2.53×10^{-2}	3.730	1.02×10^{-5}	5.623	3.62×10^{-1}	6.991	2.69×10^{-2}
1.181	1.15×10^{-3}	3.806	1.15×10^{-5}	5.669	4.46×10^{-1}	7.002	7.59×10^{-2}

1.219	2.88×10^{-1}	3.841	4.04×10^{-3}	5.686	1.58×10^{-1}	7.010	1.21×10^{-1}
1.325	2.39×10^{-5}	3.855	1.15×10^{-3}	5.696	1.73×10^{-1}	7.035	6.80×10^{-2}
1.430	5.11×10^{-3}	3.877	4.85×10^{-5}	5.707	2.08×10^{-2}	7.057	8.71×10^{-1}
1.449	2.61×10^{-2}	3.944	6.71×10^{-1}	5.718	7.80×10^{-2}	7.077	8.51×10^{-1}
1.594	6.68×10^{-5}	3.970	1.12×10^{-4}	5.761	1.70×10^{-2}	7.100	7.19×10^{-2}
1.816	8.05×10^{-3}	4.004	2.60×10^{-6}	5.785	1.16×10^{-2}	7.112	3.05×10^{-1}
1.895	1.65×10^{-1}	4.038	3.98×10^{-6}	5.804	4.04×10^{-3}	7.118	4.92×10^{-1}
1.928	4.92×10^{-3}	4.059	1.40×10^{-1}	5.824	5.57×10^{-1}	7.138	4.68×10^{-1}
1.956	1.34×10^{-1}	4.082	2.14×10^{-5}	5.840	6.19×10^{-3}	7.194	3.36×10^{-2}
2.007	2.93×10^{-3}	4.097	2.82×10^{-5}	5.916	7.20×10^{-3}	7.205	2.82×10^{-1}
2.035	4.42×10^{-7}	4.107	1.59×10^{-6}	6.047	4.29×10^{-2}	7.227	2.30×10^{-1}
2.053	7.04×10^{-5}	4.122	2.53×10^{-5}	6.074	3.75×10^{-1}	7.283	9.40×10^{-1}
2.070	2.29×10^{-3}	4.132	8.59×10^{-6}	6.102	1.65×10^{-1}	7.299	7.93×10^{-1}
2.088	3.92×10^{-2}	4.148	1.02×10^{-5}	6.117	1.98×10^{-1}	7.318	1.47×10^{-1}
2.104	3.00×10^{-1}	4.161	4.65×10^{-4}	6.129	1.38×10^{-2}	7.401	6.89×10^{-1}
2.119	4.36×10^{-5}	4.168	3.68×10^{-1}	6.141	1.44×10^{-1}	7.508	3.68×10^{-1}
2.154	4.55×10^{-3}	4.178	8.05×10^{-3}	6.161	7.19×10^{-2}	7.595	1.48×10^{-2}
2.491	4.97×10^{-2}	4.223	8.99×10^{-3}	6.193	5.08×10^{-1}	7.648	2.61×10^{-1}
2.739	3.06×10^{-2}	4.562	7.08×10^{-1}	6.228	7.64×10^{-1}	7.667	8.61×10^{-1}
2.796	3.26×10^{-2}	4.599	5.57×10^{-1}	6.249	7.55×10^{-1}	7.755	7.55×10^{-1}
2.852	8.23×10^{-2}	4.679	9.50×10^{-1}	6.299	3.62×10^{-1}	7.959	6.08×10^{-1}
2.883	1.21×10^{-1}	4.808	4.92×10^{-1}	6.346	6.80×10^{-1}	8.021	2.44×10^{-2}
2.914	1.65×10^{-1}	4.827	2.71×10^{-1}	6.386	6.35×10^{-1}	8.081	7.17×10^{-1}
2.951	4.92×10^{-1}	4.849	4.92×10^{-3}	6.410	2.07×10^{-1}	8.197	4.17×10^{-1}
3.022	4.38×10^{-1}	4.872	1.15×10^{-5}	6.438	1.12×10^{-1}	8.478	6.17×10^{-1}
3.233	8.51×10^{-1}	4.930	3.55×10^{-1}	6.461	1.47×10^{-1}		

Table S4. Buckets with corresponding *p*-values for differentiation of samples from Germany and China based on PSYCHE spectra. Significant buckets (14) with a *p*-value < 0.0016667 are highlighted.

ppm	*p*-value	ppm	*p*-value	ppm	*p*-value	ppm	*p*-value
0.885	7.94×10^{-7}	2.769	6.01×10^{-4}	3.716	6.05×10^{-6}	7.076	5.11×10^{-1}
0.955	1.04×10^{-1}	2.815	1.81×10^{-1}	3.782	2.77×10^{-5}	6.903	9.18×10^{-1}
1.263	1.08×10^{-1}	3.125	1.51×10^{-1}	4.001	2.71×10^{-7}	6.547	1.43×10^{-2}
1.339	6.75×10^{-7}	3.408	7.94×10^{-7}	4.86	2.78×10^{-8}	7.494	5.42×10^{-1}
1.578	9.44×10^{-6}	3.552	9.34×10^{-7}	5.353	5.74×10^{-1}	7.755	1.08×10^{-1}
2.049	7.94×10^{-7}	3.586	2.81×10^{-3}	5.58	7.72×10^{-2}	0.699	9.05×10^{-1}
2.242	4.42×10^{-1}	3.618	4.86×10^{-7}	6.117	9.56×10^{-1}		
2.31	5.20×10^{-6}	3.66	2.82×10^{-6}	6.339	2.70×10^{-2}		

Table S5. Buckets with corresponding *p*-values for differentiation of samples from Germany and France based on PSYCHE spectra.

ppm	*p*-value	ppm	*p*-value	ppm	*p*-value	ppm	*p*-value
0.885	5.52×10^{-2}	2.769	1.63×10^{-1}	3.716	9.07×10^{-2}	7.076	8.12×10^{-1}
0.955	2.97×10^{-1}	2.815	1.72×10^{-1}	3.782	1.26×10^{-1}	6.903	9.35×10^{-1}
1.263	2.84×10^{-1}	3.125	8.57×10^{-1}	4.001	7.92×10^{-2}	6.547	3.28×10^{-1}
1.339	6.05×10^{-2}	3.408	3.60×10^{-2}	4.86	1.15×10^{-2}	7.494	2.31×10^{-3}
1.578	9.41×10^{-2}	3.552	1.26×10^{-1}	5.353	3.72×10^{-1}	7.755	1.57×10^{-1}
2.049	6.46×10^{-2}	3.586	2.24×10^{-1}	5.58	8.26×10^{-1}	0.699	5.02×10^{-1}
2.242	2.79×10^{-1}	3.618	1.25×10^{-2}	6.117	4.31×10^{-1}		
2.31	9.07×10^{-2}	3.66	8.32×10^{-2}	6.339	8.80×10^{-1}		

Table S6. Buckets with corresponding *p*-values for differentiation of samples from China and France based on PSYCHE spectra. Significant buckets (13) with a *p*-value < 0.0016667 are highlighted.

ppm	*p*-value	ppm	*p*-value	ppm	*p*-value	ppm	*p*-value
0.885	1.71×10^{-5}	2.769	1.12×10^{-2}	3.716	1.12×10^{-4}	7.076	3.68×10^{-1}
0.955	2.21×10^{-1}	2.815	3.62×10^{-1}	3.782	7.05×10^{-4}	6.903	9.01×10^{-1}
1.263	1.94×10^{-1}	3.125	1.58×10^{-1}	4.001	3.12×10^{-6}	6.547	3.80×10^{-2}
1.339	1.21×10^{-5}	3.408	3.51×10^{-5}	4.86	1.15×10^{-5}	7.494	1.40×10^{-1}
1.578	1.95×10^{-4}	3.552	6.80×10^{-6}	5.353	8.12×10^{-1}	7.755	1.08×10^{-2}
2.049	6.04×10^{-6}	3.586	1.48×10^{-2}	5.58	6.99×10^{-2}	0.699	7.55×10^{-1}
2.242	8.81×10^{-1}	3.618	3.34×10^{-4}	6.117	6.17×10^{-1}		
2.31	1.07×10^{-4}	3.66	1.18×10^{-4}	6.339	3.80×10^{-2}		

Table S7. Buckets with corresponding *p*-values for differentiation of samples from Germany and China based on ASAP-HSQC spectra. Significant buckets (24) with a *p*-value < 0.00125 are highlighted.

ppm	*p*-value	ppm	*p*-value	ppm	*p*-value
(0.89, 14.51)	1.91×10^{-6}	(3.97, 64.81)	7.94×10^{-1}	(7.10, 110.36)	4.61×10^{-1}
(0.96, 14.67)	8.27×10^{-2}	(4.08, 65.94)	5.96×10^{-3}	(5.05, 73.27)	4.91×10^{-3}
(1.31, 23.61)	7.19×10^{-5}	(4.15, 63.32)	7.22×10^{-3}	(3.76, 62.28)	3.18×10^{-5}
(1.57, 25.95)	2.29×10^{-4}	(4.23, 60.21)	5.42×10^{-2}	(3.66, 62.24)	1.67×10^{-4}
(1.30, 32.68)	1.94×10^{-2}	(4.30, 63.20)	1.66×10^{-3}	(3.70, 63.01)	5.50×10^{-5}
(1.32, 30.51)	6.05×10^{-6}	(5.24, 70.33)	7.72×10^{-2}	(3.30, 71.23)	5.20×10^{-6}
(2.05, 28.13)	2.07×10^{-6}	(5.21, 71.62)	7.10×10^{-1}	(3.40, 73.04)	2.82×10^{-6}
(2.31, 35.02)	8.14×10^{-6}	(5.37, 93.39)	7.02×10^{-6}	(3.64, 74.49)	6.72×10^{-5}
(2.79, 26.48)	3.74×10^{-4}	(6.35, 107.21)	2.71×10^{-1}	(3.79, 74.41)	1.19×10^{-6}
(3.13, 54.81)	7.94×10^{-1}	(5.32, 129.06)	6.72×10^{-5}	(3.73, 83.72)	5.14×10^{-5}

(3.54, 67.43)	6.29×10^{-1}	(5.37, 131.04)	1.78×10^{-4}	(4.02, 79.77)	2.09×10^{-5}
(3.63, 61.42)	2.24×10^{-5}	(6.54, 108.30)	1.13×10^{-3}	(3.98, 75.46)	1.39×10^{-6}
(3.58, 64.33)	5.20×10^{-6}	(6.55, 107.46)	2.39×10^{-2}		
(3.87, 70.90)	1.90×10^{-4}	(6.60, 108.26)	4.61×10^{-1}		

Table S8. Buckets with corresponding *p*-values for differentiation of samples from Germany and France based on ASAP-HSQC spectra.

ppm	*p*-value	ppm	*p*-value	ppm	*p*-value
(0.89, 14.51)	1.19×10^{-1}	(3.97, 64.81)	8.12×10^{-1}	(7.10, 110.36)	7.50×10^{-1}
(0.96, 14.67)	8.21×10^{-1}	(4.08, 65.94)	8.43×10^{-2}	(5.05, 73.27)	3.19×10^{-1}
(1.31, 23.61)	9.64×10^{-2}	(4.15, 63.32)	3.08×10^{-1}	(3.76, 62.28)	1.73×10^{-1}
(1.57, 25.95)	3.72×10^{-1}	(4.23, 60.21)	3.66×10^{-1}	(3.66, 62.24)	1.52×10^{-1}
(1.30, 32.68)	7.06×10^{-1}	(4.30, 63.20)	2.22×10^{-1}	(3.70, 63.01)	1.52×10^{-1}
(1.32, 30.51)	6.54×10^{-2}	(5.24, 70.33)	6.56×10^{-1}	(3.30, 71.23)	7.72×10^{-2}
(2.05, 28.13)	1.51×10^{-1}	(5.21, 71.62)	1.61×10^{-1}	(3.40, 73.04)	8.64×10^{-2}
(2.31, 35.02)	2.77×10^{-2}	(5.37, 93.39)	4.44×10^{-2}	(3.64, 74.49)	1.49×10^{-1}
(2.79, 26.48)	3.08×10^{-1}	(6.35, 107.21)	4.69×10^{-1}	(3.79, 74.41)	1.12×10^{-1}
(3.13, 54.81)	4.69×10^{-1}	(5.32, 129.06)	6.29×10^{-2}	(3.73, 83.72)	7.25×10^{-2}
(3.54, 67.43)	2.89×10^{-1}	(5.37, 131.04)	5.78×10^{-1}	(4.02, 79.77)	2.09×10^{-1}
(3.63, 61.42)	5.20×10^{-1}	(6.54, 108.30)	1.43×10^{-2}	(3.98, 75.46)	1.32×10^{-1}
(3.58, 64.33)	1.63×10^{-1}	(6.55, 107.46)	9.41×10^{-2}		
(3.87, 70.90)	1.23×10^{-1}	(6.60, 108.26)	6.85×10^{-1}		

Table S9. Buckets with corresponding *p*-values for differentiation of samples from China and France based on ASAP-HSQC spectra. Significant buckets (19) with a *p*-value < 0.00125 are highlighted.

ppm	*p*-value	ppm	*p*-value	ppm	*p*-value
(0.89, 14.51)	3.32×10^{-5}	(3.97, 64.81)	7.08×10^{-1}	(7.10, 110.36)	2.77×10^{-1}
(0.96, 14.67)	6.80×10^{-2}	(4.08, 65.94)	6.08×10^{-2}	(5.05, 73.27)	2.44×10^{-2}
(1.31, 23.61)	2.19×10^{-3}	(4.15, 63.32)	3.26×10^{-2}	(3.76, 62.28)	4.88×10^{-4}
(1.57, 25.95)	6.44×10^{-4}	(4.23, 60.21)	1.34×10^{-1}	(3.66, 62.24)	4.65×10^{-4}
(1.30, 32.68)	2.87×10^{-2}	(4.30, 63.20)	7.47×10^{-3}	(3.70, 63.01)	4.65×10^{-4}
(1.32, 30.51)	6.01×10^{-5}	(5.24, 70.33)	1.12×10^{-1}	(3.30, 71.23)	5.70×10^{-5}
(2.05, 28.13)	9.10×10^{-6}	(5.21, 71.62)	1.73×10^{-1}	(3.40, 73.04)	1.52×10^{-4}
(2.31, 35.02)	1.12×10^{-4}	(5.37, 93.39)	4.88×10^{-4}	(3.64, 74.49)	8.45×10^{-4}
(2.79, 26.48)	3.59×10^{-3}	(6.35, 107.21)	5.91×10^{-1}	(3.79, 74.41)	1.81×10^{-5}
(3.13, 54.81)	9.90×10^{-1}	(5.32, 129.06)	2.11×10^{-3}	(3.73, 83.72)	1.15×10^{-3}

(3.54, 67.43)	8.81×10^{-1}	(5.37, 131.04)	2.38×10^{-4}	(4.02, 79.77)	1.95×10^{-4}
(3.63, 61.42)	9.10×10^{-6}	(6.54, 108.30)	4.55×10^{-2}	(3.98, 75.46)	1.91×10^{-5}
(3.58, 64.33)	3.32×10^{-5}	(6.55, 107.46)	1.10×10^{-1}		
(3.87, 70.90)	5.31×10^{-3}	(6.60, 108.26)	2.61×10^{-1}		

Table S10. Comparison of the accuracies of classification models based on the mid-polar extract and the polar extraction method from previous studies [32].

**model**	**accuracy ($MeOD/CD_3CN$)**	**accuracy ($MeOD/D_2O$)**
CN/DE	95.9% (±0.8%)	96.6% (±0.6%)
DE/FR	83.4% (±2.0%)	92.0% (±1.8%)
CN/FR	93.7% (±1.1%)	92.6% (±1.2%)

Table S11. Information about walnut samples used for the study (supplier, declared geographical origin, harvest year, variety).

supplier	country	declared region	declared origin	harvest year	variety	sample
priv. person no. 1	China	Xinjiang	unknown	2017	unknown	17-CN-001
priv. person no. 2	China	unknown	unknown	2017	unknown	17-CN-002
CRP Food Import Export GmbH	China	Xinjiang	unknown	2017	Tulare	17-CN-005
CRP Food Import Export GmbH	China	Xinjiang	unknown	2018	unknown	18-CN-007
CRP Food Import Export GmbH	China	Xinjiang	unknown	2018	unknown	18-CN-008
CRP Food Import Export GmbH	China	Xinjiang	unknown	2018	Chandler	18-CN-009
CRP Food Import Export GmbH	China	Shanxi	unknown	2018	unknown	18-CN-010
CRP Food Import Export GmbH	China	Yunnan	unknown	2018	unknown	18-CN-011
CRP Food Import Export GmbH	China	Yunnan	unknown	2018	unknown	18-CN-012
CRP Food Import Export GmbH	China	Yunnan	unknown	2018	unknown	18-CN-013
CRP Food Import Export GmbH	China	unknown	unknown	2019	Tulare	19-CN-014
CRP Food Import Export GmbH	China	Shanxi	unknown	2019	unknown	19-CN-015
CRP Food Import Export GmbH	China	unknown	unknown	2019	Chandler	19-CN-016
CRP Food Import Export GmbH	China	Xinjiang	unknown	2019	unknown	19-CN-017
CRP Food Import Export GmbH	China	Yunnan	unknown	2019	unknown	19-CN-018
Isemarkt Hamburg	Germany	Niedersachsen	Altes Land	2017	unknown	17-DE-005
Isemarkt Hamburg	Germany	Niedersachsen	Stade	2017	unknown	17-DE-006
Isemarkt Hamburg	Germany	Niedersachsen	Ostyork	2017	unknown	17-DE-007
Isemarkt Hamburg	Germany	Baden-Württemberg	Baden	2017	unknown	17-DE-008
priv. person no. 3	Germany	Nordrhein-Westfalen	Petershagen	2017	unknown	17-DE-010
priv. person no. 4	Germany	Schleswig-Holstein	Wedel	2018	unknown	18-DE-012
priv. person no. 3	Germany	Nordrhein-Westfalen	Petershagen	2018	unknown	18-DE-013
priv. person no. 3	Germany	Nordrhein-Westfalen	Petershagen	2018	unknown	18-DE-014
Lochwald-Riednuss GbR	Germany	Hessen	Biebesheim	2018	Lara	18-DE-015

supplier	country	declared region	declared origin	harvest year	variety	sample
Lochwald-Riednuss GbR	Germany	Hessen	Biebesheim	2018	Weinsberg 1	18-DE-016
Lochwald-Riednuss GbR	Germany	Hessen	Biebesheim	2018	Mars	18-DE-017
Lochwald-Riednuss GbR	Germany	Hessen	Biebesheim	2018	Seifersdorfer Runde	18-DE-018
Lochwald-Riednuss GbR	Germany	Hessen	Biebesheim	2018	Weidenheimer 139	18-DE-019
Lochwald-Riednuss GbR	Germany	Hessen	Biebesheim	2018	Hartley	18-DE-020
Lochwald-Riednuss GbR	Germany	Hessen	Biebesheim	2018	Franquette	18-DE-021
Baumschule Matthias Schott	Germany	Baden-Württemberg	Sasbach-Leiselheim	2018	Geisenheimer 139	18-DE-022
Baumschule Matthias Schott	Germany	Baden-Württemberg	Sasbach-Leiselheim	2018	Geisenheimer 286	18-DE-023
Baumschule Matthias Schott	Germany	Baden-Württemberg	Sasbach-Leiselheim	2018	Geisenheimer 120	18-DE-024
Baumschule Matthias Schott	Germany	Baden-Württemberg	Sasbach-Leiselheim	2018	Geisenheimer 1247	18-DE-025
Baumschule Matthias Schott	Germany	Baden-Württemberg	Sasbach-Leiselheim	2018	Geisenheimer 26	18-DE-026
Baumschule Matthias Schott	Germany	Baden-Württemberg	Sasbach-Leiselheim	2018	Esterhazy II	18-DE-027
Baumschule Matthias Schott	Germany	Baden-Württemberg	Sasbach-Leiselheim	2018	Geisenheimer 1239	18-DE-028
Baumschule Matthias Schott	Germany	Baden-Württemberg	Sasbach-Leiselheim	2018	Weinsberg 1	18-DE-029
priv. person no. 5	Germany	Sachsen-Anhalt	Wulferstedt	2018	unknown	18-DE-030
priv. person no. 6	Germany	Niedersachsen	Celle	2018	unknown	18-DE-031
priv. person no. 7	Germany	Niedersachsen	Westerbeck	2018	unknown	18-DE-032
Dennis Prigge Obstbau	Germany	Niedersachsen	Jork	2019	unknown	19-DE-033
priv. person no. 7	Germany	Niedersachsen	Westerbeck	2019	unknown	19-DE-034
priv. person no. 3	Germany	Nordrhein-Westfalen	Petershagen	2019	unknown	19-DE-035
priv. person no. 3	Germany	Nordrhein-Westfalen	Petershagen	2019	unknown	19-DE-036
Baumschule Matthias Schott	Germany	Baden-Württemberg	Sasbach-Leiselheim	2019	Esterhazy II	19-DE-037
Baumschule Matthias Schott	Germany	Baden-Württemberg	Sasbach-Leiselheim	2019	Geisenheimer Nr. 139	19-DE-038
Baumschule Matthias Schott	Germany	Baden-Württemberg	Sasbach-Leiselheim	2019	Geisenheimer Nr. 286	19-DE-039
Baumschule Matthias Schott	Germany	Baden-Württemberg	Sasbach-Leiselheim	2019	Geisenheimer Nr 120	19-DE-040

supplier	country	declared region	declared origin	harvest year	variety	sample
Baumschule Matthias Schott	Germany	Baden-Württemberg	Sasbach-Leiselheim	2019	Geisenheimer Nr. 1239	19-DE-041
Baumschule Matthias Schott	Germany	Baden-Württemberg	Sasbach-Leiselheim	2019	Geisenheimer Nr. 26	19-DE-042
Baumschule Matthias Schott	Germany	Baden-Württemberg	Sasbach-Leiselheim	2019	unknown	19-DE-043
Lochwald-Riednuss GbR	Germany	Hessen	Biebesheim	2019	Geisenheim Nr. 26	19-DE-045
Lochwald-Riednuss GbR	Germany	Hessen	Biebesheim	2019	Geisenheim Nr. 138	19-DE-046
Lochwald-Riednuss GbR	Germany	Hessen	Biebesheim	2019	Martlog	19-DE-047
Lochwald-Riednuss GbR	Germany	Hessen	Biebesheim	2019	Milotai 10	19-DE-048
Lochwald-Riednuss GbR	Germany	Hessen	Biebesheim	2019	Esterhazy II	19-DE-049
Lochwald-Riednuss GbR	Germany	Hessen	Biebesheim	2019	Mars	19-DE-050
Lochwald-Riednuss GbR	Germany	Hessen	Biebesheim	2019	Lara	19-DE-051
priv. person no. 8	Germany	Nordrhein-Westfalen	Harsewinkel	2019	unknown	19-DE-052
priv. person no. 8	Germany	Nordrhein-Westfalen	Herzebrock-Clarholz	2019	unknown	19-DE-053
priv. person no. 8	Germany	Niedersachsen	Bad Laer	2019	unknown	19-DE-054
priv. person no. 8	Germany	Nordrhein-Westfalen	Gütersloh	2019	unknown	19-DE-055
priv. person no. 9	Germany	Niedersachsen	Ottersberg	2019	unknown	19-DE-056
Coopenoix	France	Auvergne-Rhône-Alpes	Isere/Drome/Savoie	2016	unknown	16-FR-002
Delphinoix	France	Auvergne-Rhône-Alpes	Isere/Drome/Savoie	2017	unknown	17-FR-003
Coopenoix	France	Auvergne-Rhône-Alpes	Isere/Drome/Savoie	2017	Lara	17-FR-004
SCA Unicoque	France	Auvergne-Rhône-Alpes	Allier	2017	Franquette	17-FR-008
SCA Unicoque	France	Nouvelle-Aquitaine	Gironde	2017	Lara	17-FR-009
SCA Unicoque	France	Nouvelle-Aquitaine	Charente	2017	Fernor	17-FR-010
SCA Unicoque	France	Nouvelle-Aquitaine	Deux-Sèvres	2017	Lara	17-FR-011
SCA Unicoque	France	Pays de la Loire	Sarthe	2017	Franquette	17-FR-012
SCA Unicoque	France	Occitanie	Aude	2017	Franquette	17-FR-013
SCA Unicoque	France	Occitanie	Tarn-et-Garonne	2017	Lara	17-FR-014

supplier	country	declared region	declared origin	harvest year	variety	sample
SCA Unicoque	France	Nouvelle-Aquitaine	Gironde	2017	Fernor	17-FR-015
SCA Unicoque	France	Pays de la Loire	Sarthe	2017	Lara	17-FR-016
SCA Unicoque	France	Auvergne-Rhône-Alpes	Allier	2017	Lara	17-FR-017
SCA Unicoque	France	Occitanie	Aude	2017	Lara	17-FR-018
SCA Unicoque	France	Nouvelle-Aquitaine	Gironde	2017	Franquette	17-FR-019
SCA Unicoque	France	Nouvelle-Aquitaine	Lot-et-Garonne	2017	Lara	17-FR-020
SCA Unicoque	France	Nouvelle-Aquitaine	Charente	2017	Lara	17-FR-021
SCA Unicoque	France	Nouvelle-Aquitaine	Charente	2017	Franquette	17-FR-022
SCA Unicoque	France	Occitanie	Gers	2017	Lara	17-FR-023
SCA Unicoque	France	Nouvelle-Aquitaine	Lot-et-Garonne	2017	Fernor	17-FR-024
Nuss-Baumschule Gubler GmbH AG	France	unknown	unknown	2017	Franquette	17-FR-025
Nuss-Baumschule Gubler GmbH AG	France	unknown	unknown	2017	Lara	17-FR-026
Nuss-Baumschule Gubler GmbH AG	France	unknown	unknown	2017	Fernor	17-FR-027
nutwork Handelsgesellschaft mbH	France	unknown	unknown	2018	unknown	18-FR-028
SCA Unicoque	France	Auvergne-Rhône-Alpes	Allier	2018	Lara	18-FR-030
SCA Unicoque	France	Nouvelle-Aquitaine	Gironde	2018	Lara	18-FR-031
SCA Unicoque	France	Nouvelle-Aquitaine	Charente	2018	Lara	18-FR-032
SCA Unicoque	France	Occitanie	Aude	2018	Lara	18-FR-033
SCA Unicoque	France	Occitanie	Gers	2018	Lara	18-FR-034
SCA Unicoque	France	Pays de la Loire	Sarthe	2018	Lara	18-FR-035
SCA Unicoque	France	Nouvelle-Aquitaine	Deux-Sèvres	2018	Lara	18-FR-036

supplier	country	declared region	declared origin	harvest year	variety	sample
SCA Unicoque	France	Nouvelle-Aquitaine	Charente	2018	Fernor	18-FR-037
SCA Unicoque	France	Auvergne-Rhône-Alpes	Allier	2018	Franquette	18-FR-038
SCA Unicoque	France	Nouvelle-Aquitaine	Charente	2018	Franquette	18-FR-039
SCA Unicoque	France	Occitanie	Aude	2018	Franquette	18-FR-040
SCA Unicoque	France	Pays de la Loire	Sarthe	2018	Franquette	18-FR-041
Intermarché	France	Dordogne	unknown	2018	Franquette	18-FR-043
priv. person no. 10	France	Provence-Alpes-Côte d'Azur	Cogolin	2019	unknown	19-FR-044
Lochwald-Riednuss GbR	France	Isère	unknown	2019	Lara	19-FR-045
SCA Unicoque	France	Occitanie	Aude	2019	Lara	19-FR-046
SCA Unicoque	France	Occitanie	Gers	2019	Lara	19-FR-047
SCA Unicoque	France	Nouvelle-Aquitaine	Gironde	2019	Lara	19-FR-048
SCA Unicoque	France	Nouvelle-Aquitaine	Lot-et-Garonne	2019	Lara	19-FR-049
SCA Unicoque	France	unknown	unknown	2019	Lara	19-FR-050
SCA Unicoque	France	Nouvelle-Aquitaine	Gironde	2019	Franquette	19-FR-051
SCA Unicoque	France	Occitanie	Aude	2019	Franquette	19-FR-052
SCA Unicoque	France	Pays de la Loire	Sarthe	2019	Franquette	19-FR-053
SCA Unicoque	France	Nouvelle-Aquitaine	Charente-Maritime	2019	Lara	19-FR-054
SCA Unicoque	France	Nouvelle-Aquitaine	Charente	2019	Franquette	19-FR-055
SCA Unicoque	France	Nouvelle-Aquitaine	Lot-et-Garonne	2019	Fernor	19-FR-056
SCA Unicoque	France	Auvergne-Rhône-Alpes	Allier	2019	Lara	19-FR-057
SCA Unicoque	France	Occitanie	Tarn-et-Garonne	2019	Lara	19-FR-058
SCA Unicoque	France	Nouvelle-Aquitaine	Charente	2019	Fernor	19-FR-059
SCA Unicoque	France	Nouvelle-Aquitaine	Charente-Maritime	2019	Franquette	19-FR-060
SCA Unicoque	France	Nouvelle-Aquitaine	Gironde	2019	Fernor	19-FR-061

supplier	country	declared region	declared origin	harvest year	variety	sample
SCA Unicoque	France	Pays de la Loire	Sarthe	2019	Lara	19-FR-062
SCA Unicoque	France	Auvergne-Rhône-Alpes	Allier	2019	Franquette	19-FR-063
SCA Unicoque	France	Nouvelle-Aquitaine	Deux-Sèvres	2019	Lara	19-FR-064
SCA Unicoque	France	Nouvelle-Aquitaine	Charente	2019	Lara	19-FR-065
nutwork Handelsgesellschaft mbH	France	unknown	unknown	2019	unknown	19-FR-066
Rieser Nuss GmbH & Co. KG	France	unknown	unknown	2019	Franquette	19-FR-067
Rieser Nuss GmbH & Co. KG	France	unknown	unknown	2019	Franquette	19-FR-068
Rieser Nuss GmbH & Co. KG	France	unknown	unknown	2019	unknown	19-FR-069
Rieser Nuss GmbH & Co. KG	France	unknown	unknown	2019	unknown	19-FR-070

S1. Variable sized buckets (183) used for the classification of the different two-class models using the 1D ^{1}H NOESY spectra.

[ppm]: 0.7213 – 0.6978; 0.747 – 0.7383; 1.414 – 1.2365; 0.5691 – 0.545; 0.3637 – 0.3354; 0.8144 – 0.7845; 0.849 – 0.8343; 0.9299 – 0.8676; 0.9415 – 0.9306; 0.9928 – 0.9418; 1.0098 – 0.9928; 1.0397 – 1.0288; 1.1087 – 1.0904; 1.1212 – 1.1081; 1.4365 – 1.4236; 1.4538 – 1.4448; 1.6602 – 1.5273; 1.8326 – 1.8001; 1.9054 – 1.8842; 1.9379 – 1.918; 1.9709 – 1.9417; 2.0098 – 2.0043; 2.1715 – 2.1356; 2.5529 – 2.429; 2.7481 – 2.7291; 2.8437 – 2.7493; 2.8594 – 2.8444; 2.8903 – 2.8755; 2.9249 – 2.9028; 3.2395 – 3.2257; 3.2745 – 3.2642; 3.3323 – 3.3108; 3.3368 – 3.3326; 3.357 – 3.3377; 3.3669 – 3.358; 3.3769 – 3.3673; 3.435 – 3.41; 3.4786 – 3.4501; 3.5024 – 3.4902; 3.5162 – 3.5037; 3.5486 – 3.5162; 3.5692 – 3.5489; 3.6026 – 3.5753; 3.6221 – 3.6026; 3.834 – 3.7785; 3.8475 – 3.834; 3.8619 – 3.8475; 3.8914 – 3.8626; 3.9778 – 3.9621; 4.0484 – 4.0285; 4.0699 – 4.0491; 4.0908 – 4.0725; 4.103 – 4.0914; 4.1097 – 4.1036; 4.1251 – 4.1187; 4.1393 – 4.1255; 4.1556 – 4.1396; 4.1669 – 4.1556; 4.1704 – 4.1665; 4.1852 – 4.1707; 4.8153 – 4.8012; 4.8381 – 4.8166; 4.8599 – 4.8381; 4.884 – 4.8602; 5.1889 – 5.0345; 5.2997 – 5.2843; 5.2859 – 5.2756; 5.2762 – 5.2608; 5.2618 – 5.2528; 5.2534 – 5.2361; 5.3263 – 5.3003; 5.3937 – 5.3658; 5.4101 – 5.3944; 5.4233 – 5.4111; 5.4505 – 5.4277; 5.4749 – 5.4515; 5.4977 – 5.4749; 5.5414 – 5.5199; 5.6008 – 5.5683; 5.6412 – 5.6046; 5.6765 – 5.6621; 5.691 – 5.6813; 5.7006 – 5.691; 5.7128 – 5.7019; 5.7234 – 5.7128; 5.7654 – 5.7558; 5.8094 – 5.7991; 5.847 – 5.8338; 5.7946 – 5.7751; 5.8325 – 5.8152; 5.9237 – 5.9086; 6.054 – 6.0392; 6.0852 – 6.062; 6.1131 – 6.0912; 6.1205 – 6.1134; 6.1368 – 6.1205; 6.1997 – 6.1869; 6.2344 – 6.2216; 6.2562 – 6.2424; 6.6164 – 6.5939; 6.6334 – 6.6177; 6.6806 – 6.6591; 6.3753 – 6.3166; 6.3959 – 6.3753; 6.4238 – 6.3965; 6.4514 – 6.4251; 6.4716 – 6.4514; 6.522 – 6.472; 6.5288 – 6.5224; 6.5744 – 6.5294; 6.5936 – 6.5744; 6.7878 – 6.7734; 6.7641 – 6.7564; 6.7422 – 6.7307; 6.7207 – 6.7121; 6.8681 – 6.7968; 6.9971 – 6.9849; 6.9721 – 6.9631; 7.0042 – 6.9994; 7.0125 – 7.0067; 7.0514 – 7.018; 7.0629 – 7.051; 7.1098 – 7.0902; 7.1146 – 7.1101; 7.1197 – 7.1159; 7.0889 – 7.0642; 7.1483 – 7.1287; 7.1981 – 7.1891; 7.2115 – 7.1993; 7.2423 – 7.2122; 7.322 – 7.3139; 7.3046 – 7.2937; 7.2911 – 7.2751; 7.538 – 7.4783; 7.6568 – 7.6401; 7.6728 – 7.6613; 7.598 – 7.5913; 7.7887 – 7.7222; 8.0863 – 8.0757; 8.0272 – 8.015; 7.9928 – 7.9254; 8.2041 – 8.1906; 8.5273 – 8.4294; 3.9627 – 3.9261; 4.571 – 4.5527; 4.6275 – 4.571; 4.6994 – 4.6577; 5.0313 – 4.9755; 5.2358 – 5.1867; 6.3172 – 6.281; 7.4199 – 7.3813; 0.8272 – 0.8169; 0.8632 – 0.8551; 1.0285 – 1.0111; 1.0583 – 1.0461; 1.1492 – 1.1431; 1.1671 – 1.1591; 1.1835 – 1.1781; 1.2243 – 1.2137; 2.0441 – 2.0268; 2.0608 – 2.0444; 2.0788 – 2.0608; 2.0964 – 2.0788; 2.1109 – 2.0964; 2.1272 – 2.1109; 2.9734 – 2.9281; 3.0681 – 2.9753; 3.4103 – 3.3904; 3.4494 – 3.435; 3.4864 – 3.4799; 3.7785 – 3.6825; 3.6822 – 3.6257; 4.0288 – 3.9791; 4.2587 – 4.1877; 4.9723 – 4.8881; 5.3658 – 5.327; 6.1766 – 6.1458; 6.1426 – 6.1384; 6.6893 – 6.6857; 6.8931 – 6.879; 6.9066 – 6.8937; 6.9281 – 6.9069; 6.9403 – 6.9281.

S2. Variable sized buckets (30) for the classification of different two-class models using the PSYCHE spectra.

[ppm]: 0.6796 – 0.7186; 0.8553 – 0.9139; 0.9383 – 0.9725; 1.2360 – 1.2897; 1.2946 – 1.3825; 1.5289 – 1.6265; 2.0024 – 2.0951; 2.2220 – 2.2611; 2.2709 – 2.3490; 2.7443 – 2.7932; 2.7932 – 2.8371; 3.0714 – 3.1788; 3.3838 – 3.4326; 3.5302 – 3.5741; 3.5741 – 3.5986; 3.6034 – 3.6327; 3.6376 – 3.6815; 3.6864 – 3.7450; 3.7450 – 3.8182; 3.9744 – 4.0281; 4.8384 – 4.8823; 5.2923 – 5.4144; 5.5608 – 5.5999; 6.0880 – 6.1466; 6.3028 – 6.3760; 6.5175 – 6.5761; 6.8641 – 6.9422; 7.0301 – 7.1228; 7.4791 – 7.5084; 7.7281 – 7.7818.

S3. Variable sized buckets (40) used for the classification of different two-class models using the ASAP-HSQC spectra.

[ppm] (^{13}C) x [ppm] (^{1}H): (14.1020 – 14.9081) x (0.8373 – 0.9394); (14.2632 – 15.0694) x (0.9340 – 0.9931); (23.1309 – 24.0983) x (1.2455 – 1.3691); (25.4688 – 26.4362) x (1.4872 – 1.6537); (25.9525 – 27.0005) x (2.7226 – 2.8586); (27.5648 – 28.6934) x (1.9706 – 2.1210); (29.5802 – 31.4344) x (1.2133 – 1.4228); (32.0793 – 33.2886) x (1.2402 – 1.3583); (34.3366 – 35.7070) x (2.2392 – 2.3735); (54.2487 – 55.3773) x (3.0824 – 3.1848); (59.7306 – 60.6980) x (4.1942 – 4.2587); (61.1011 – 61.7460) x (3.6088 – 3.6518); (61.8373 – 62.7241) x (3.7268 – 3.8019); (61.8371 – 62.6435) x (3.6193 – 3.7053); (62.4822 – 63.5302) x (3.6516 – 3.7429); (62.5522 – 63.8420) x (4.2641 – 4.3446); (62.7134 – 63.9226) x (4.1029 – 4.2050); (63.8420 – 64.8094) x (3.5121 – 3.6571); (64.4063 – 65.2125) x (3.9311 – 4.0063); (65.6156 – 66.2605) x (4.0600 – 4.0976); (66.9860 – 67.8728) x (3.5067 – 3.5766); (69.9688 – 70.6944) x (5.2040 – 5.2738);

(70.5331 – 71.2587) x (3.8505 – 3.8881); (70.7051 – 71.7531) x (3.2380 – 3.3561); (71.2587 – 71.9842) x (5.1772 – 5.2362); (72.5592 – 73.5266) x (3.3347 – 3.4582); (72.9516 – 73.5965) x (5.0214 – 5.0805); (73.9297 – 74.8971) x (3.7575 – 3.8395); (74.0103 – 74.9777) x (3.5656 – 3.7053); (74.9777 – 75.9451) x (3.9309 – 4.0329); (79.2503 – 80.2984) x (3.9738 – 4.0598); (83.3618 – 84.0873) x (3.6784 – 3.7751); (92.8637 – 93.9118) x (5.3329 – 5.4081); (106.8103 – 107.6165) x (6.3051 – 6.4018); (106.9715 – 107.9389) x (6.5253 – 6.5790); (107.9389 – 108.6645) x (6.5092 – 6.5629); (107.9389 – 108.5839) x (6.5790 – 6.6220); (109.8737 – 110.8411) x (7.0624 – 7.1322); (128.4154 – 129.7052) x (5.2523 – 5.3866); (130.4308 – 131.6400) x (5.3007 – 5.4350).

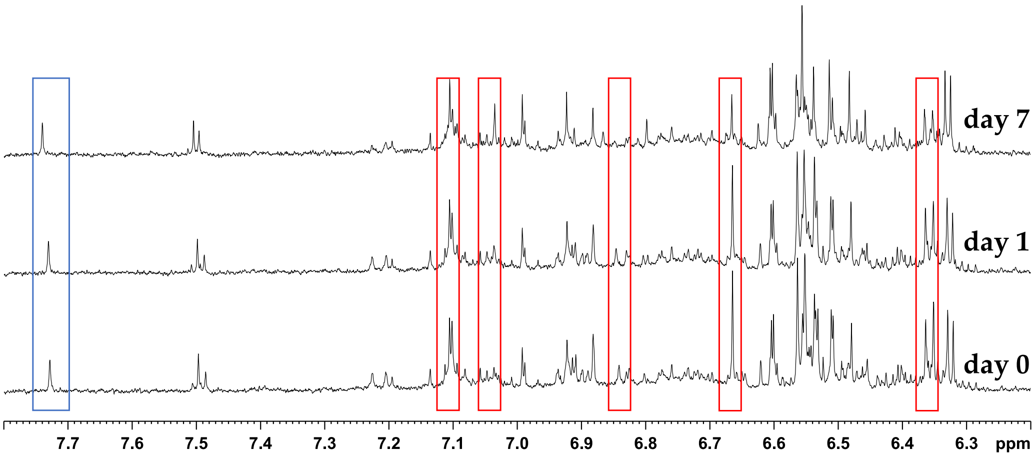

Figure S1. Aromatic region of ^{1}H NOESY spectra (400 MHz, NS = 128) of the stability measurement (day 0 - day 7) of the acetonitrile-d_3/methanol-d_4 (method A) extract of a representative walnut sample. An example of a signal shift is the singlet marked in blue, which was detected at 7.73 ppm in the spectrum acquired on the day of extraction, but showed an altered chemical shift of 7.74 ppm after 7 days. Selected areas indicate exemplary changes in signal intensities (red).

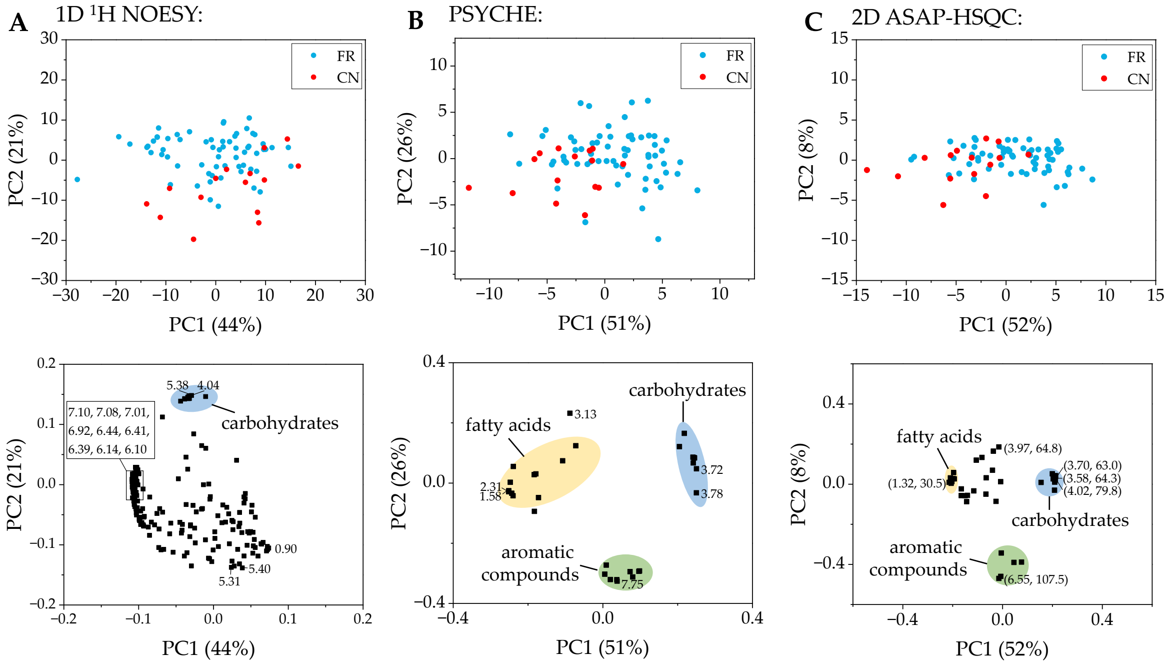

Figure S2. PCA score and loading plot of the differentiation of French and Chinese walnut samples using the different NMR experiments. **A:** Results using the 1D ¹H NOESY spectra. Explained variance: PC1 = 44%, PC2 = 21%. **B:** Results using the PSYCHE spectra. Explained variance: PC1 = 51%, PC2 = 26%. **C:** Results using the 2D ASAP-HSQC spectra. Explained variance: PC1 = 52%, PC2 = 8%. FR: France, CN: China.

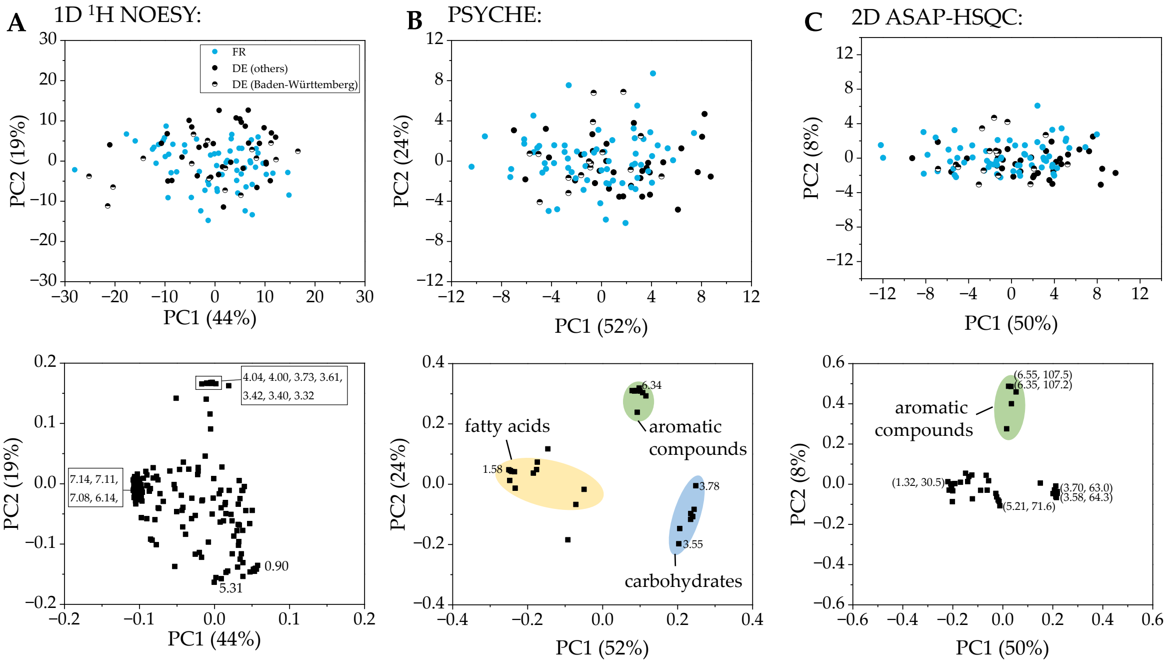

Figure S3. PCA score and loading plot of the differentiation of French and German walnut samples using the different NMR experiments. German samples from Baden-Württemberg are highlighted. **A:** Results using the 1D ¹H NOESY spectra. Explained variance: PC1 = 44%, PC2 = 19%. **B:** Results using the PSYCHE spectra. Explained variance: PC1 = 52%, PC2 = 24%. **C:** Results using the 2D ASAP-HSQC spectra. Explained variance: PC1 = 50%, PC2 = 8%. DE: Germany, FR: France.

1D ^{1}H NOESY | **PSYCHE** | **ASAP-HSQC**

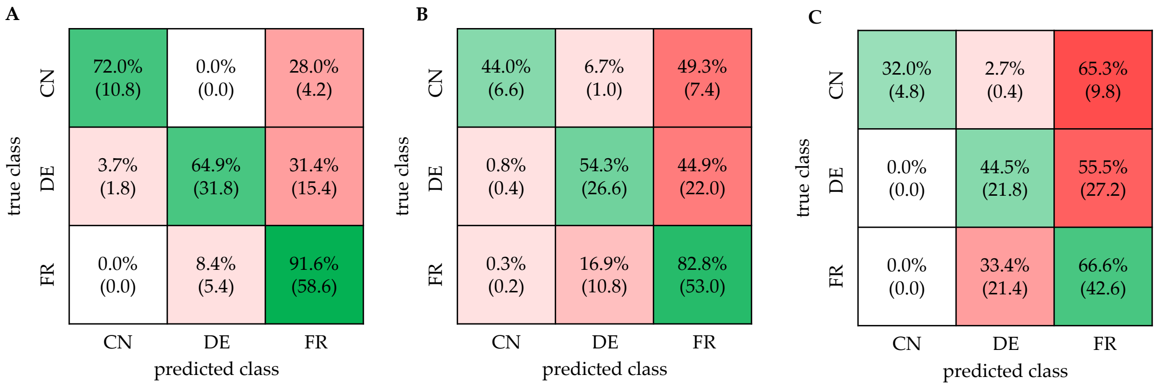

	CN	DE	FR
sensitivity	72.0%	64.9%	91.6%
specificity	98.4%	93.2%	69.4%
accuracy	79.1% (± 1.6%)		

	CN	DE	FR
sensitivity	44.0%	54.3%	82.8%
specificity	99.5%	85.1%	54.1%
accuracy	67.3% (± 1.6%)		

	CN	DE	FR
sensitivity	32.0%	44.5%	66.6%
specificity	100%	72.4%	42.2%
accuracy	54.1% (± 1.7%)		

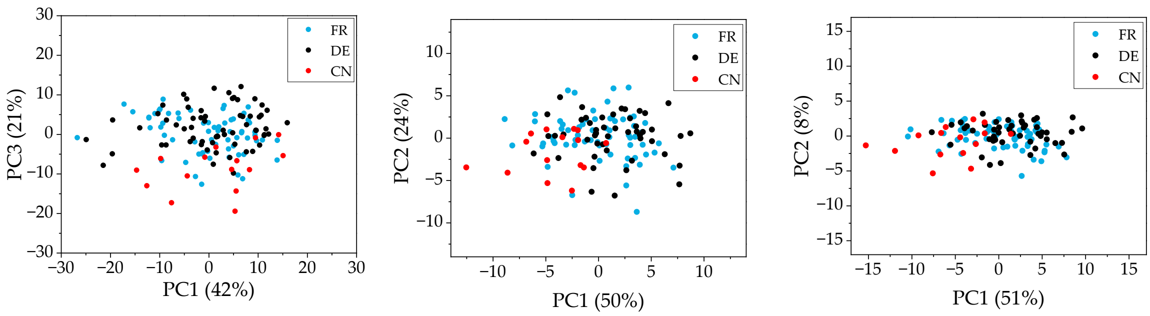

Figure S4. Confusion matrices of the three-class models based on the analysis of each kind of NMR spectra using a linear support vector machine algorithm. CN: China, DE: Germany, FR: France. Accuracies: A: 79.1% (± 1.6%); B: 67.3% (± 1.6%); C: 54.1% (± 1.7%). The PCA score plots of the different NMR spectra are shown below.

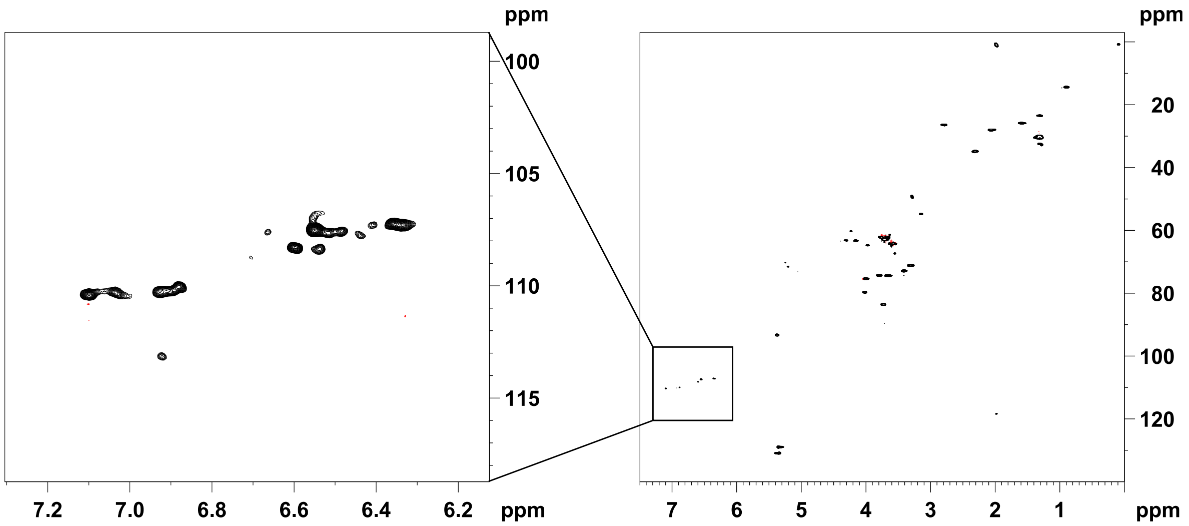

Figure S5. ASAP-HSQC spectrum (400 MHz) of a walnut extract (acetonitrile/methanol, method B) acquired with 256 scans resulting in a total acquisition time of 4 hours and 19 minutes. The region of the aromatic signals is shown expanded.
